# A multilocus phylogeny of the fish genus *Poeciliopsis*: Solving taxonomic uncertainties and preliminary evidence of reticulation

**DOI:** 10.1002/ece3.4874

**Published:** 2019-01-25

**Authors:** Mariana Mateos, Omar Domínguez‐Domínguez, Alejandro Varela‐Romero

**Affiliations:** ^1^ Department of Wildlife and Fisheries Sciences Texas A&M University College Station Texas; ^2^ Laboratorio de Biología Acuática, Facultad de Biología Universidad Michoacana de San Nicolás de Hidalgo Morelia Michoacán Mexico; ^3^ Departamento de Investigaciones Científicas y Tecnológicas Universidad de Sonora Hermosillo Sonora Mexico

**Keywords:** biogeography, hybridization, phylogenetic networks, reticulation, species trees, Trans‐Mexican Volcanic Belt

## Abstract

The fish genus *Poeciliopsis* constitutes a valuable research system for evolutionary ecology, whose phylogenetic relationships have not been fully elucidated. We conducted a multilocus phylogenetic study of the genus based on seven nuclear and two mitochondrial loci with a thorough set of analytical approaches, that is, concatenated (also known as super‐matrix), species trees, and phylogenetic networks. Although several relationships remain unresolved, the overall results uncovered phylogenetic affinities among several members of this genus*. *A population previously considered of undetermined taxonomic status could be unequivocally assigned to *P. scarlli*; revealing a relatively recent dispersal event across the Trans‐Mexican Volcanic Belt (TMVB) or Pacific Ocean, which constitute a strong barrier to north–south dispersal of many terrestrial and freshwater taxa. The closest relatives of *P. balsas*, a species distributed south of the TMVB, are distributed in the north; representing an additional north–south split in the genus. An undescribed species of *Poeciliopsis*, with a highly restricted distribution (i.e., a short stretch of the Rio Concepcion; just south of the US‐Mexico border), falls within the *Leptorhaphis* species complex. Our results are inconsistent with the hypothesis that this species originated by “breakdown” of an asexual hybrid lineage. On the other hand, network analyses suggest one or more possible cases of reticulation within the genus that require further evaluation with genome‐wide marker representation and additional analytical tools. The most strongly supported case of reticulation occurred within the subgenus *Aulophallus* (restricted to Central America), and implies a hybrid origin for *P. retropinna* (i.e., between *P. paucimaculata* and *P. elongata*). We consider that *P. balsas* and *P.* new species are of conservation concern.

## 1 INTRODUCTION

**Figure 1:**
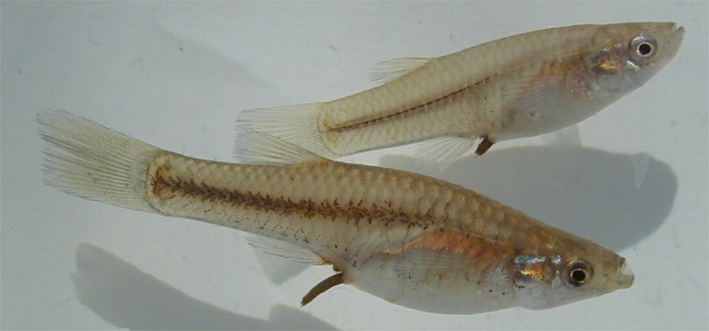
Unidentified females of *Poeciliopsis* from the Rio Concepcion, Sonora, Mexico. This drainage is inhabited by two sexual species (*Poeciliopsis occidentalis* and an undescribed species referred to as “*Poeciliopsis* new species” in the present study), and two hybrid biotypes (*Poeciliopsis monacha*‐*occidentalis* and “*Poeciliopsis monacha*‐new species”). Photograph by Robert C. Vrijenhoek

The freshwater livebearing fish genus *Poeciliopsis* (Poeciliidae; Figure 1) has served as an invaluable study system for several evolutionary ecology questions, including unisexual reproduction and the maintenance of sex (reviewed in Schlupp & Riesch, 2011); life history (e.g., placentation) and intergenomic conflict (Jue, Foley, Reznick, O'Neill, & O'Neill, 2018; O'Neill et al., 2007; Pires, McBride, & Reznick, 2007; Reznick, Mateos, & Springer, 2002; Zúñiga‐Vega, Reznick, & Johnson, 2007); and historical biogeography of Mexico and Central America (Beltrán‐Lopez, Domínguez‐Domínguez, Pérez‐Rodríguez, Piller, & Doadrio, 2018; Larson, Scarborough, Sayre, & Johnson, 2012; Mateos, Sanjur, & Vrijenhoek, 2002). A robust phylogenetic framework of the genus would greatly enhance its usefulness for macroevolutionary research. Notwithstanding, the phylogenetic relationships within *Poeciliopsis* and the taxonomic status of several populations remain uncertain because of poor resolution of past analyses, lack of specimens, the sole use of mitochondrial makers (Mateos et al., 2002), or the use of limited analytical tools (e.g., concatenated vs. species trees methods; Pollux, Meredith, Springer, Garland, & Reznick, 2014).

**Figure 2:**
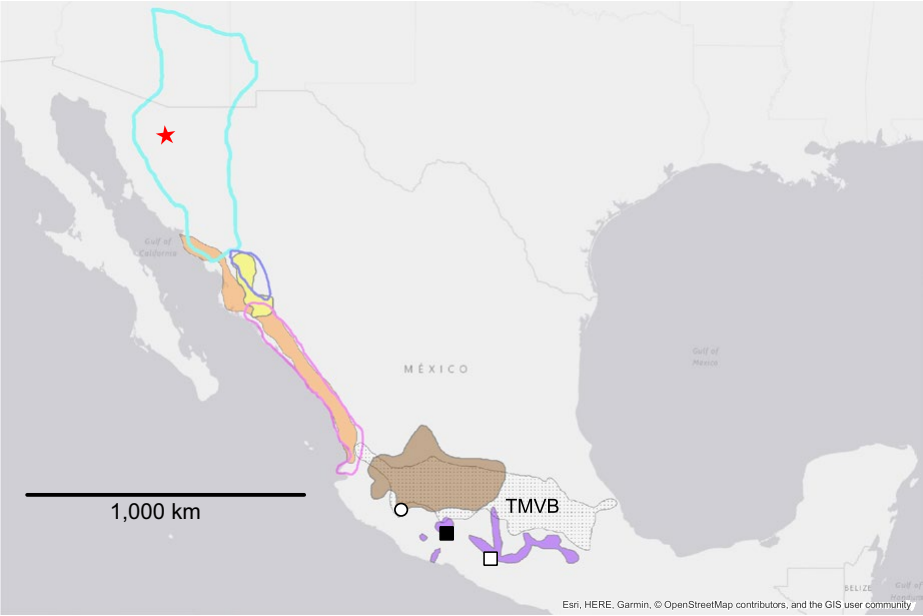
Distribution of the Predominantly Northern clade (clade E). Colors and symbols correspond to those used in Figure 6. Stippled polygon labeled TMVB represents the location of the Trans‐Mexican Volcanic Belt. The other polygons indicate distribution of *P. balsas* (violet filled), *P. occidentalis s.l.* (i.e., *P. occidentalis s.s.* + *P. sonoriensis*; turquoise border), *P. prolifica* (orange filled), *P. infans* (brown filled), *P. lucida* (yellow filled), *P. viriosa* (magenta border), and *P. monacha* (blue border). Red star =sampling location (and geographic range, at this scale) of *P.* new species. Black square =sampling location of *P. balsas*. The polygons for *P. balsas* connect known records of occurrence, including a report for the Cutzamala sub‐basin (at ca. 19°30′N, 100°30′W; in 1985–1986) by Paulo‐Maya and Ramirez‐Enciso (1997), and a 2017 report of occurrence in Ajuchitan del Progreso, Guerrero (empty square; see Supporting information Table S4). The empty circle indicates a locality (Laguna Zapotlán) where *P. infans* was reported up until 1998. A 2016 survey of this locality did not find *P. infans*. Instead, *P. prolifica*, likely introduced, were highly abundant (ODD, personal observation). Map generated in ArcMap v.10.5.1 (Environmental Systems Research Institute, Inc)

**Figure 3:**
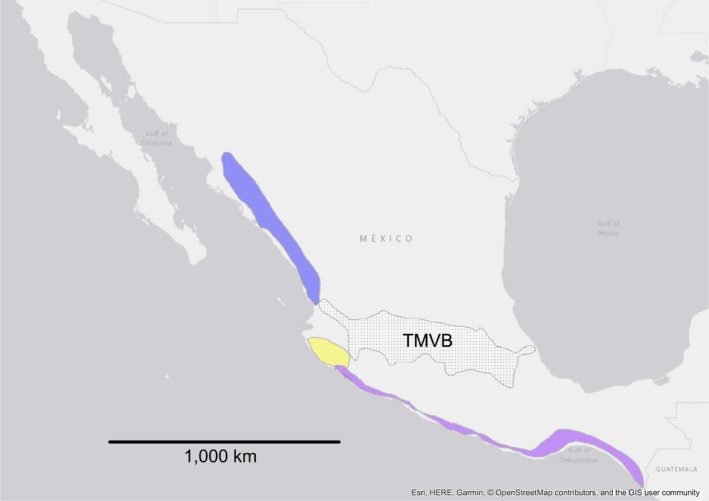
Distribution of clade M. Stippled polygon labeled TMVB represents the location of the Trans‐Mexican Volcanic Belt. The other filled polygons indicate distribution of *P. latidens* (blue), *P. fasciata* (violet), and *P. baenschi* (yellow). Colors correspond to those used in Figure 6. Map generated in ArcMap v.10.5.1 (Environmental Systems Research Institute, Inc)

**Figure 4:**
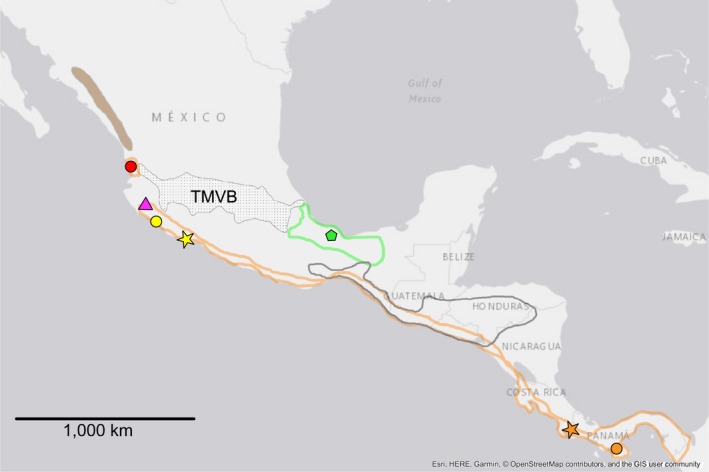
Distribution of the clade K. Stippled polygon labeled TMVB represents the location of the Trans‐Mexican Volcanic Belt. Colors and symbols correspond to those used in Figure 6. The other polygons indicate distribution of *P. presidionis *(brown filled), *P. gracilis* (green border), *P. pleurospilus* (gray bordered; not examined in this study), *P. turrubarensis *+ *P. scarlli* (orange bordered). The geographical limits between the sister taxa *P. scarlli* and *P. turrubarensis* have not been identified; their respective type localities are indicated by a yellow and an orange star. Filled circles represent sampling localities for *P. scarlli* (red and yellow) and *P. turrubarensis* (orange). Magenta triangle = sampling location (and geographic range, at this scale) of *P. turneri*. Green pentagon = sampling location (and geographic range, at this scale) of *P. catemaco*. Map generated in ArcMap v.10.5.1 (Environmental Systems Research Institute, Inc)

The genus *Poeciliopsis* is distributed from southern Arizona to Colombia (Rosen & Bailey, 1963) and is comprised of 24 valid species (Eschmeyer, Fricke, & Laan, 2018), divided into two subgenera (*Poeciliopsis* and *Aulophallus*). Within this range, the Trans‐Mexican Volcanic Belt (TMVB; a massively uplifted and geologically active physiographic feature that crosses central Mexico from west to east; Figures 2, 3, 4) is considered a current or historical dispersal barrier for many freshwater organisms (Agorreta et al., 2013; Domínguez‐Domínguez, Doadrio, & Pérez‐Ponce de León, 2006; Huidobro, Morrone, Villalobos, & Álvarez, 2006; Hulsey, León, Johnson, Hendrickson, & Near, 2004; Mateos, 2005; Mulcahy & Mendelson, 2000; Mulcahy, Morrill, & Mendelson, 2006; Obregón‐Barboza, Contreras‐Balderas, & Lourdes Lozano‐Vilano, 1994; Ornelas‐García, Domínguez‐Domínguez, & Doadrio, 2008; Parra‐Olea, Windfield, Velo‐Antón, & Zamudio, 2012; Pedraza‐Lara, Doadrio, Breinholt, & Crandall, 2012; Pérez‐Rodríguez, Domínguez‐Domínguez, Pérez Ponce de León, & Doadrio., 2009), including *Poeciliopsis *(Mateos et al., 2002). With the exception of *Poeciliopsis infans *(brown polygon in Figure 2), which inhabits drainages on the TMVB, no species of *Poeciliopsis* appeared to traverse this region, and several species have their southern or northern distribution limits bordering the TMVB (Figures 2, 3, 4).

A previous study (Mateos et al., 2002) examined the historical biogeography of most of the nominal species in the genus *Poeciliopsis* based on two mitochondrial genes, and concluded that at least three north versus south divergence events in the area of the TMVB contributed to the diversification of this genus. Nonetheless, that study was unable to resolve several relationships and phylogeographic patterns within the genus. Herein, we attempted to further resolve them by adding several nuclear genes, taxa, and analytical tools that can account for reticulation (i.e., phylogenetic network analyses).

## 2 MATERIALS AND METHODS

**Figure 5:**
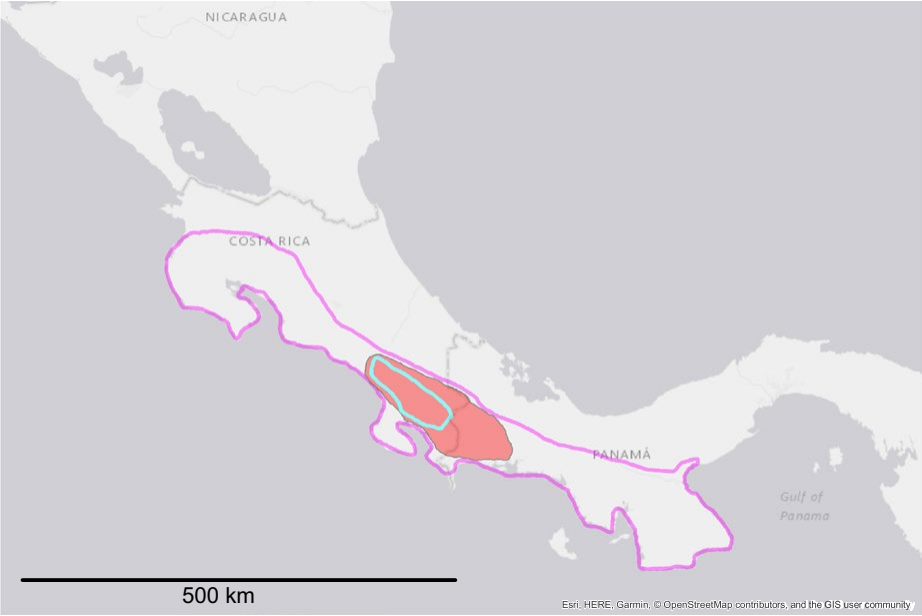
Distribution of the subgenus *Aulophallus*. Colored polygons indicate distribution of *P. elongata *(magenta border), *P. retropinna* (red filled), and *P. paucimaculata* (turquoise border). Colors correspond to those used in Figure 6. Map generated in ArcMap v.10.5.1 (Environmental Systems Research Institute, Inc)

We examined the majority of the species of the subgenera *Poeciliopsis* and *Aulophallus*. The latter subgenus is comprised of three species and is restricted to Central America (Figure 5). We used one specimen per valid species of *Poeciliopsis* (following Miller, Minckley, & Norris, 2005), excluding: *P. sonoriensis *(a close relative of *P. occidentalis *sensu* stricto*; hereafter we refer to *P. sonoriensis* + *P. occidentalis s.s*. as “*P. occidentalis s.l*.”); and three members of the *gracilis* complex [*P. pleurospilus*, *P. hnilickai*, and the recently described *P. santaelena* (Bussing, 2008)]. For *P. scarlli*, which was synonymized with *P. turrubarensis* by Miller et al. (2005) despite relatively deep mitochondrial divergences (Mateos et al., 2002), we included one specimen from north of the TMVB (representing a population of unknown status; sensu Miller et al., 2005), as well as a specimen from south of the TMVB examined by (Mateos et al., 2002; see Figure 4). We also included two taxa not examined by Mateos et al. (2002): *P. balsas* (from the Balsas drainage; south of the TMVB; purple polygon and black square in Figure 2); and undescribed species of *Poeciliopsis* (hereafter “*P*. new species”; red star in Figure 2) endemic to a short stretch of the Rio Concepcion (also known as Rio Magdalena) in Sonora, Mexico. The majority of sequences were retrieved from GenBank (Supporting information Table S1) as part of two studies (Mateos et al., 2002; Pollux et al., 2014). For analyses that employ outgroup rooting (i.e., the concatenated analyses), the tree was rooted at the branch joining the two subgenera, a relationship that is supported by examination of taxa outside the genus *Poeciliopsis* (Mateos et al., 2002).

We used primers and PCR conditions listed in Mateos et al. (2002) for the mitochondrial Cytb and ND2 genes, and in Meredith, Pires, Reznick, and Springer (2010) for the nuclear genes: X_src; RH; Rag1; Myh6; SH3PX3; Glyt; and ENC1. PCR amplicons were directly sequenced with the Sanger method on an ABI 3730 DNA Analyzer at the University of Arizona Genetics Core (UAGC) in both directions with the PCR primers. The forward and reverse strands of each amplicon were assembled and edited within Sequencer v.4.8. (Genecodes, Ann Arbor, MI, USA).

### 2.1 Alignments and datasets

We used MAFFT v.7 (Katoh, Rozewicki, & Yamada, 2017) to align sequences of each gene separately (strategy = Auto). All alignments were visually inspected in Mesquite v3.10 (Maddison & Maddison, 2018) for unexpected gaps and were translated to amino acid sequences to verify the absence of early stop codons. Phylogenetic analyses were performed on three datasets: the mitochondrial Cytb and the ND2 genes (“MT only”), the seven nuclear genes (“Nuclear only”), all mitochondrial and nuclear genes together “All genes”.

### 2.2 Standard phylogenetic analyses

Unless otherwise noted, all phylogenetic analyses were performed on the CIPRES server (Miller, Pfeiffer, & Schwartz, 2010). We conducted two types of traditional (i.e., non‐network) phylogenetic analyses: concatenated and species trees. The concatenated analyses (or super‐matrix) assume that all genes in a dataset share the same genealogy. The species trees analyses (as implemented in *BEAST v.2.4.7; Bouckaert et al., 2014; Heled & Drummond, 2010) utilize a coalescence‐based approach and assume that each linkage group (in our case, each nuclear gene and the mtDNA linkage group), share the same species tree, but not the same gene genealogy.

To identify the best substitution models among candidate models, we used jModeltest v. 2.1.10 v20160303 (Darriba, Taboada, Doallo, & Posada, 2012) and PartitionFinder v.2 (Lanfear, Frandsen, Wright, Senfeld, & Calcott, 2017). For PartitionFinder, we defined codon positions and introns (when appropriate; see Dataset S4) to search for the best partitioning scheme and substitution model per partition.

We performed the following concatenated analyses and associated clade support measures: RaxML v.8.2.10 rapid bootstrap (Stamatakis, 2014); IQ‐TREE v.1.6.2 [http://iqtree.cibiv.univie.ac.at/; SH‐aLRT, aBayes, and Ultrafast bootstrap (Hoang, Chernomor, Haeseler, Minh, & Vinh, 2018; Trifinopoulos, Nguyen, Haeseler, & Minh, 2016)], PhyML v.20120412 [http://www.atgc-montpellier.fr/phyml/; bootstrap, SH‐aLRT, and aBayes; (Guindon et al., 2010)]; and MrBayes v3.2.6 (Ronquist et al., 2012). We assumed both a single partition and the best partitioning scheme suggested by PartitionFinder2 (details in Supporting information Table S2); except for PhyML, as it only runs single partition analyses. A majority rule consensus tree of each analysis (if not summarized directly by program) was obtained with SumTrees v.4.1.0, which is part of Dendropy (Sukumaran & Holder, 2010), and visualized with FigTree v.1.4.3pre (available from http://tree.bio.ed.ac.uk/software/figtree/). Bayesian analyses ran four independent MCMC chains. Sampled posterior distributions from MrBayes and *BEAST analyses were examined in Tracer v.1.7‐Pre20171127 (available from http://github.com/beast-dev/tracer/) and used to assess convergence on stable posterior probabilities, determine adequate burnin, and ensure effective samples sizes (ESS) greater than 200. The following analyses obtained with RWTY (Warren, Geneva, & Lanfear, 2017) were also used to assess convergence and adequate sampling of the posterior: split frequency comparisons; average standard deviation of split frequency (ASDSF) plot; approximate ESS of tree topologies.

### 2.3 Phylogenetic network analyses

To assess whether one or more of the poorly resolved relationships could be better explained by hybridization rather than incomplete lineage sorting (ILS), we performed the network analyses implemented in PhyloNetworks (Solís‐Lemus & Ané, 2016; Solís‐Lemus, Bastide, & Ané, 2017). Briefly, we ran Bucky v.1.4.4 (Ané, Larget, Baum, Smith, & Rokas, 2007; Larget, Kotha, Dewey, & Ané, 2010) assuming an alpha of 1, and using as input the posterior tree samples of a partitioned MrBayes run where all the parameters (including topology) were unlinked among the eight linkage groups. The output was processed according to the “TICR pipeline” (Stenz, Larget, Baum, & Ané, 2015; see Dataset S5 or http://crsl4.github.io/PhyloNetworks.jl/latest/man/ticr_howtogetQuartetCFs/#TICR-pipeline-1) to generate the quartet concordance factors. We ran SNaQ for a maximum number of hybrid egdes (hmax) from 0 to 4, with several starting trees, including the species tree (scripts and files available in Dataset S5). The best network at each level that could be rooted correctly (i.e., at the branch joining the subgenera *Poeciliopsis* and *Aulophallus*) was retained and visualized with the PhyloPlots (https://github.com/cecileane/PhyloPlots.jl) and Dendroscope v.3.5.9 (Huson et al., 2007; Huson & Scornavacca, 2012). Pseudolikelihood values (in negative log likelihood units) and corresponding Akaike Information Criterion (AIC = −2(Pseudolikelihood) + 2*k*, where *k = *number of hybrid edges) scores were compared for each retained tree/network. For the tree (0 hybrid edges) and each network, we fitted the quartet CFs (command “fittedQuartetCF”) and plotted them against the observed quartet CF values to determine whether the observed gene tree discordance (quartet CFs) is better fitted by one of the networks (i.e., points closer to the diagonal). Although other tools are available for distinguishing ILS versus reticulation (reviewed by Folk, Soltis, Soltis, & Guralnick, 2018), our taxon sampling (i.e., one sample per species) is generally inadequate for them.

## 3 RESULTS

New sequences have been deposited under GenBank Accession Numbers: MH118098‐MH118114 (Supporting information Table S1). The alignment containing all genes is deposited as Dataset S1. No unexpected indels or early stop codons were detected. The substitution models and partitioning schemes selected by jModeltest and PartitionFinder are shown in Supporting information Table S2. The implemented models are detailed in Supporting information Tables S2 and S3 (when a particular program could not accommodate the model, the next more complex model available was used). The XML files used for *BEAST are deposited as Datasets S2 and S3.

### 3.1 Standard phylogenetic analyses

**Figure 6:**
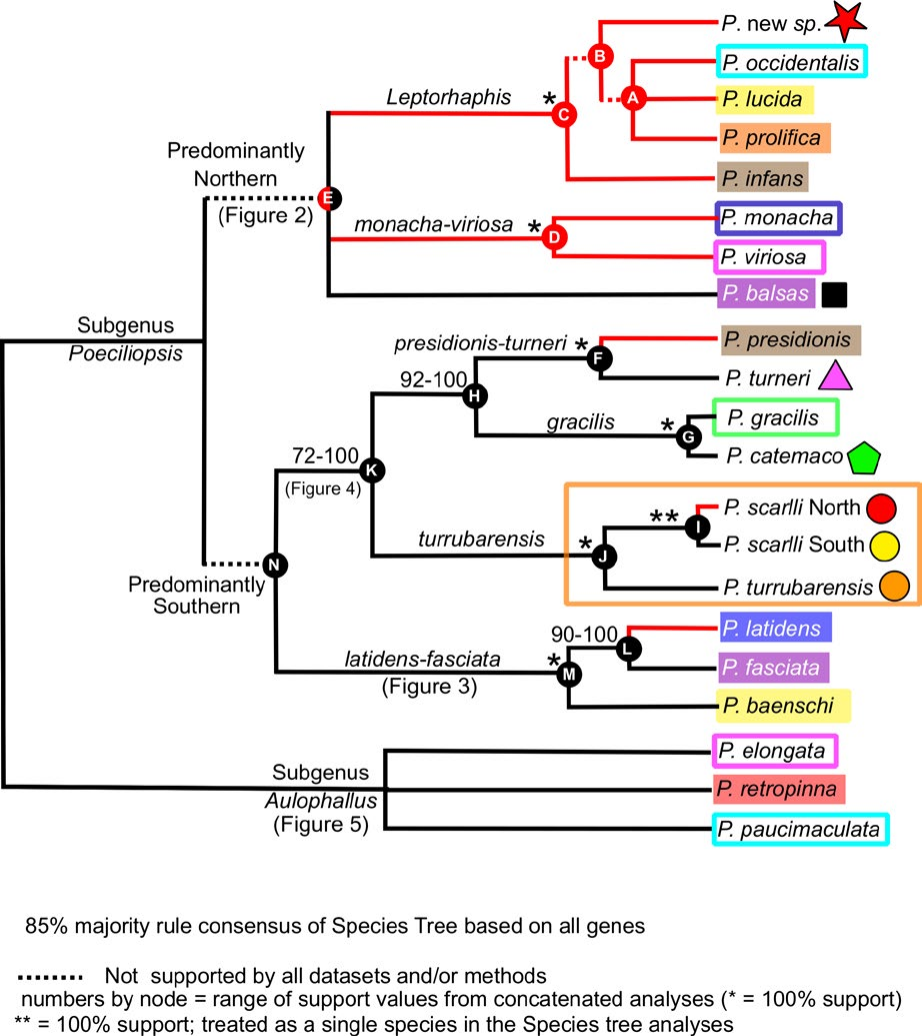
Species Tree based on the “All genes” dataset. About 85% Majority rule consensus tree. Support values at nodes represent the range of values obtained from the concatenated analyses (detailed in Supporting information Table S3) or * if support was 100%. Dashed internal branches indicate that some analyses or methods recovered <50% support. The two *P. scarlli* branches, which were treated as members of the same species in the species trees analyses, were manually drawn based on the concatenated analyses. Black versus red branches, respectively, indicate that the lineage's native distribution is south versus north of (or on) the Trans‐Mexican Volcanic Belt (TMVB). Node letters are used in the main text, figures, and Supporting information Table S3. Color of node label indicates most parsimonious distribution of that ancestor with regard to the TMVB (red = north; black = south; red/black = equivocal). Symbols by taxon labels, and filled/empty colored boxes surrounding taxon labels correspond to sampling localities or distribution polygons in Figures 2, 3, 4, 5.

**Figure 7:**
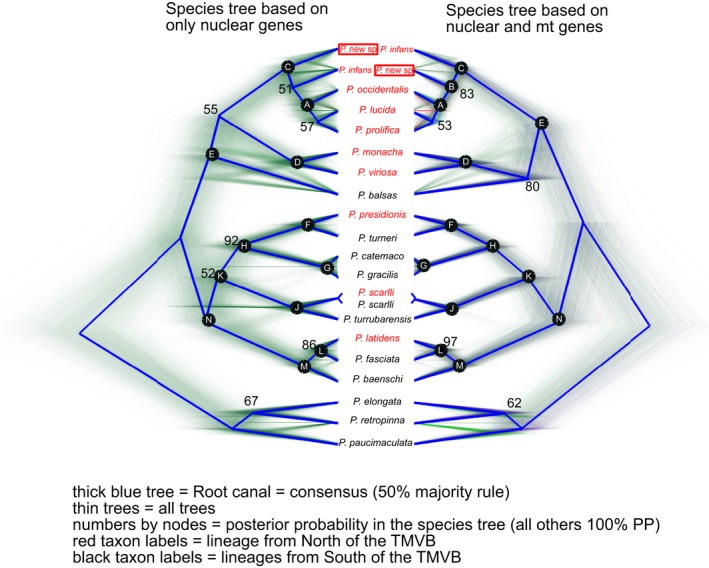
Comparison of Species Trees based on the “Nuclear only” (left) and “All genes” (right) datasets. Densitree depiction of the posterior distribution of trees for each analysis (thin trees); thick blue trees (i.e., “root canal”) represent a 50% majority rule consensus. Numbers adjacent to nodes are posterior probabilities (PP); lack of a number indicates 100% PP. The two *P. scarlli* terminal branches were manually drawn based on the concatenated analyses. Nodes labels correspond to those used in main text, figures, and Supporting information Table S3. Black versus red terminal taxon labels, respectively, indicate that the native distribution is south versus north of (or on) the Trans‐Mexican Volcanic Belt (TMVB). Note the different position of *P.* new species (red box) and *P. infans* between the left‐ and right‐hand trees.

Figure 6 depicts the 85% majority rule consensus tree of the species tree analysis of the “All genes” dataset. Under this clade posterior probability threshold, these analyses failed to resolve relationships within three clades (clade A, clade E, and subgenus *Aulophallus*); depicted as trichotomies. In addition, several branches (depicted by dashed lines) indicate relationships that were not consistently supported by all methods and datasets (clade support values for each dataset and method are indicated in Supporting information Table S3). To further illustrate the discrepancy among datasets, Figure 7 depicts the species trees (thick blue lines) from the “Nuclear only” and “All Genes” datasets, as well as a DensiTree v.2.2.6 (Bouckaert & Heled, 2014) depiction of the species trees sampled from the posterior distribution.

Examination of the nuclear genes only (and in combination with the mitochondrial genes) yielded relationships consistent with those based on only mitochondrial genes with a few exceptions (outlined below). All analyses consistently recovered with 100% clade support the position of *P.* new sp. within the *Leptorhaphis* complex (clade C; Figures 6 and 7; Supporting information Table S3). Relationships within *Leptorhaphis*, however, were not well resolved (clade support values in green font in Supporting information Table S3). Clade A (*P. occidentalis* + *P. lucida* + *P. prolifica*; Figures 6 and 7) was supported by the two species trees analyses (98% All genes; 75% Nuclear only), and by all the concatenated analyses that included the mitochondrial genes. Most concatenated analyses of only the nuclear genes supported the alternative relationship *P. infans *+ *P. lucida* + *P. prolifica*. Similarly, all analyses (species trees and concatenated) that included the mitochondrial genes supported the sister relationship between Clade A and *P. *new sp.; that is, *P. infans* appeared as sister to the rest of *Leptorhaphis*. In contrast, all analyses of the nuclear genes only (species tree and concatenated) placed *P.* new sp. as sister to the rest of the *Leptorhaphis*, albeit with some low clade support values (e.g., 51%).

Most analyses supported the “Predominantly Northern” clade (E; Figures 6 and 7): *Leptorhaphis* (C) + *monacha*‐*viriosa* (D) + *P. balsas*. An alternative relationship (D + *P. balsas* + N) was recovered with low support (56%) in a single analysis: MrBayes partitioned analysis of MT Only (Supporting information Table S3; pink cells and blue font rows). Relationships within the “Predominantly Northern” clade exhibited stronger conflicts (Figure 7): the species trees analyses of all genes supported D + *P. balsas *(80%), whereas the species trees of only the nuclear genes supported C + D instead (55%). To trichotomy at the base of the “Predominantly Northern” clade (Figure 6) reflects this conflict.

The “Predominantly Southern” clade (clade N = K + M; Figures 6 and 7) was recovered with 100% support in both species trees analyses. It was also consistently recovered in the concatenated analyses of the nuclear genes (64%–100% support; see blue cells and orange font in Supporting information Table S3) and in the majority of the “All genes” analyses (<50%–99% support; see purple cells in Supporting information Table S3). In contrast, clade N was only recovered in a subset of the concatenated analyses of mitochondrial genes only (pink cells in Supporting information Table S3; consistent with the study of Mateos et al. (2002).

Within the subgenus *Aulophallus*, although the species trees supported the sister relationship of *P. elongata* and *P. retropinna*, clade support was low (<68%, thereby it was collapsed in Figure 6). Concatenated analyses including the mitochondrial genes supported the alternative relationship *P. retropinna* + *P. paucimaculata*. Support for these two conflicting clades varied according to model and method in the concatenated analyses of the “Nuclear only” dataset, as well as based on the individual gene examined (see red font rows in Supporting information Table S3). The fact that the individual gene analyses recovered one of these two relationships with at least 72% support, and none recovered the alternative relationship *P. paucimaculata* + *P. elongata* suggests that *P. retropinna* might have introgressed alleles from one or both of these species, potentially reflecting a case of hybrid speciation. The conflict is also evident in Densitree graphs (Figure 7), where quite a few of the posterior probability trees connect *P. retropinna* to *P. paucimaculata*. This phenomenon is further explored with the network analyses below.

### 3.2 Phylogenetic network analyses

There was substantial improvement in pseudolikelihood and AIC score in the comparison of 0 hybrid edges (“net0” = −27,530.18) versus one hybrid edge (“best_net1” = −18,390.36; Supporting information Figure S1). The hybrid edge connected the M and G lineages, where the G lineage appears to be the recipient of gene flow from the M lineage (i.e., major edge gamma = 81% and minor edge gamma = 19%; Supporting information Figure S2A). The Dendroscope depiction (which does not take into account gamma values) of the same network (Supporting information Figure S2B) shows the G lineage as hybrid of F and M. This hybrid edge and associated gamma values were consistently found in the best networks evaluated by SNaQ at all hmax = 1–4 (Supporting information Figures S2–S4). Clade M is found in Pacific slope drainages of Mexico (north and south of the TMVB; Figure 3), whereas the G clade is found in Atlantic (*P. gracilis* and *P. catemaco*; green‐bordered polygon and pentagon, respectively, in Figure 4) and Pacific (*P. pleurospilus*; not included in our analyses; white‐bordered polygon in Figure 4) slope drainages of southern Mexico. Members of both clades overlap in the area of the Isthmus of Tehuantepec (i.e., the narrowest part of Mexico between the Pacific and the Gulf of Mexico). Therefore, it appears geographically plausible that the ancestors of both clades exchanged genetic material via hybridization.

Addition of a second hybrid edge did not result in a substantial improvement. Two networks of this level were retained (Supporting information Figure S2C and D = −16,743.64; and Supporting information Figure S3A and B = −17,861.31). The first one used the network in Supporting information Figure S2A as a starting tree, and added a hybrid edge that involved members of clade C (*Leptorhaphis* complex), which exhibit a large degree of conflict among genes and analyses (see species trees; Figures 6 and 7). The second network used as a starting tree, a network with a hybrid edge connecting members of the subgenus *Aulophallus* (generated manually). The analyses retained this hybrid edge and added the hybrid edge connecting lineages G and M (as in Supporting information Figure S2A and B).

The analyses with three hybrid edges (hmax = 3; starting tree = Supporting information Figure S3A) resulted in further pseudolikelihood improvement (−12,082.5; Supporting information Figure S3C, D), but one of the hybrid edges in this network seems geographically implausible (red edges), as it connected the ancestor of the subgenus *Aulophallus* (restricted to southern Central America; Figure 6) to *P. viriosa *(restricted to north of the TMVB; magenta‐bordered polygon in Figure 2); that is, two lineages that are presently separated by ~2,500 km. The best network of the analyses with four hybrid edges (hmax = 4; starting tree = Supporting information Figure S3A) did not improve the pseudolikelihood (−12,611.73; Supporting information Figure S4C, D), and also included a hybrid edge (*Aulophallus* to lineage D; *P. viriosa* + *P. monacha*) that seems geographically implausible for the aforementioned reason. Upon manually removing this hybrid edge from this network and optimizing parameters on it (i.e., command “topologyMaxQPseudolik!”), we obtained a pseudolikelihood value of −16,833.12 (Supporting information Figure S4A, B). The three edges in this network appear to be geographically plausible, and include the G–M hybrid edge recovered in all network analyses, the hybrid edge involving *P. retropinna* (*Aulophallus*; discussed below), and a hybrid edge within clade C involving *P.* new species.

Although some networks exhibited substantial pseudolikelihood improvement (e.g., one vs. zero hybrid edges; Supporting information Figure S1), plotting the fitted versus observed quartet CFs did not reveal strong improvement of fit for any of the networks, as compared to the topology with no hybrid edges (*R* ^2^ increased from 0.973 to a maximum of 0.987; Supporting information Figure S5). This may be a consequence of high variation in the quartet concordance factors, stemming from the small number of genes (=8 linkage groups) examined. Furthermore, the small number of genes precludes the use of the bootstrap procedure to assess support for the inferred hybrid edges (and their direction). This bootstrap procedure resamples posterior distributions of gene trees (e.g., their posterior distributions generated by MrBayes for each gene) to generate the bootstrap replicates (see https://github.com/crsl4/PhyloNetworks.jl/wiki/Bootstrap-analysis).

Despite the low number of genes, the pattern within *Aulophallus* is strongly suggestive of reticulation. The concordance factor (CF) for *P. retropinna* + *P. paucimaculata* was 46.8%, whereas the CF for *P. retropinna* + *P. elongata* was 49.4% (red rows in Supporting information Table S3). This can be interpreted as effectively half of the genes supporting the former relationship and the half of the genes supporting the latter. None of the genes (or analyses) supported *P. elongate *+ *P. paucimaculata. *Assuming a null hypothesis of no reticulation, the ILS model with maximum likelihood has one tree (the major one) with probability 4/8 = 0.5, and the other 2 (minor) trees with probability each (4 + 0)/8/2 = 0.25. We used R (code and output in “testAullophalus.html”; DataDryad 10.5061/dryad.s0b85c2) to perform a simulation (*n* = 100,000) of eight gene trees according to these probabilities. The simulated distribution revealed that the probability of observing a difference in the number gene trees (for the two minor trees) of 4 (or larger) under the ILS model is 0.035 (*p*‐value). Therefore, despite the low number of genes, there appears to be strong evidence of reticulation within the subgenus *Aulophallus*.

Because the subgenus *Aulophallus* forms a clade with 100% concordance, the three possible reticulation scenarios involving *P. retropinna*, *P. elongata,* and *P. paucimaculata* cannot be fully determined on the basis of quartets only (Solís‐Lemus & Ané, 2016). All three scenarios have the same unrooted network, but differ in the direction of the various edges. In one scenario, *P. paucimaculata *is the recipient of gene flow from *P. retropinna *(or it is a hybrid between an unsampled species and *P. retropinna*)*. *In a second scenario, *P. elongata *is a hybrid between an unsampled species and *P. retropinna*. In the third scenario, *P. retropinna *is the recipient of gene flow from *P. paucimaculata* (or it is a hybrid between *P. elongata *and *P. paucimaculata*). This third scenario appears more likely because it does not require the existence of an unsampled species.

## 4 DISCUSSION

The addition of nuclear markers and taxa resulted in only a modest improvement of phylogenetic resolution, as greater support was obtained for the monophyly of the “Predominantly Southern” clade (N; Figures 6 and 7). The remaining relationships among previously studied taxa generally did not change. Lack of resolution may reflect hard polytomies, limited phylogenetic signal, incomplete lineage sorting, or reticulation. Reticulation has been inferred in other poeciliid genera (Bagley et al., 2015; Cui et al., 2013), and poeciliids are notorious for hybridizing (Rosenthal & García de León, 2010). The present data are insufficient to adequately test reticulation, but the pattern of conflict observed in subgenus *Aulophallus* strongly suggests that *P. retropinna* has an admixed genome (and possibly a genome of hybrid origin). Based on node depth in the species trees and in the individual gene trees (not shown), the *P. retropinna* lineage is relatively old, with its speciation event apparently occurring shortly after the split between *P. elongata* and *P. paucimaculata*. Geographically, this scenario seems quite plausible, as the present range of the three species overlaps (Figure 5). The geographic overlap of *P. retropinna* and its putative parental lineages (*P. elongata* and *P. paucimaculata*) suggests that reproductive isolation is effective, but further research is needed to uncover the underlying mechanisms. Below we discuss the biogeographic, taxonomic, and conservation implications of our results.

### 4.1 Historical biogeography

The Mateos et al. (2002) study lacked *P. balsas*, the only member of the genus native to the Balsas drainage, which borders the southern edge of TMVB (Figure 2). The results of the present study place *P. balsas* within the Predominantly Northern clade (clade E). Nevertheless, we are unable to tell with certainty whether *P. balsas* is sister to the remaining members of such clade (i.e., C + D monophyly), or whether it is more closely related to clade D. The alternative relationship, C + *P. balsas*, was not supported by any analyses (CF =12.4%; not shown). Although analyses of more genes may help resolve this relationship, it is also possible that the speciation events leading to the three lineages were effectively concurrent (i.e., representing a hard polytomy). This uncertainty precludes inference of ancestral distribution (e.g., north or south of the TMVB), and of the order of north versus south divergences within the Predominantly Northern clade. Nonetheless, if we assume that the species trees (Figure 7) depict relative divergence times accurately, it appears that the split between *P. balsas* and its closest relative (likely distributed within or north of the TMVB) predates the north–south splits observed within in the Predominantly Southern clade N (i.e., nodes F, I, and L; Figure 6 or 7) and may represent the earliest TMVB‐related divergence in the genus.

Our results indicate that the *Poeciliopsis* population of “undetermined taxonomic status” (sensu Miller et al., 2005) in the region immediately north of the TMVB (i.e., the area of San Blas; red circle in Figures 4 and 6) that was assigned to *P. presidionis *by Meyer, Riehl, Dawes, and Dibble (1985) and in several museum records (e.g., UMMZ 173,770 and 173,776; collected in 1958) is closely allied with *P. scarlli* specimens found immediately south (yellow circle in Figures 4 and 6) of the TMVB (uncorrected p distance = 0.73% at the mitochondrial genes; and 0.04% at the nuclear genes). Therefore, we tentatively assign the San Blas population to *P. scarlli*. This north versus south genetic divergence is 9–10 times shallower than that observed in the other two north–south species pairs of the Predominantly Southern clade: *P. presidionis* versus *P. turneri* (clade F; 7.3% at mt genes and 0.57% at nuclear genes); and *P. latidens* versus *P. fasciata* (clade L: 6.6% at mt genes and 0.47% at nuclear genes). Thus, exchange of *P. scarlli* north and south of the TMVB likely occurred relatively recently, possibly through coastal stream capture (despite a narrow continental shelf; see Mateos et al., 2002), marine dispersal, or pre‐1958 human introduction. The San Blas habitat where *P. scarlli* is found is brackish, implying that this species has relatively high tolerance to salinity, a feature shared with *P. turrubarensis* from Costa Rica (Larson et al., 2012). Although the present pattern is more consistent with a southern *P. scarlli* ancestor (node I; Figure 6) that invaded the north recently, a comprehensive population genetics study throughout the distribution of *P. scarlli* and *P. turrubarensis* is necessary to better infer the direction and timing of exchange (see Taxonomic Considerations). Prior to the present study, the geographic distributions and phylogenetic relationships of *Poeciliopsis* and *Poecilia* (Mateos, 2005; Mateos et al., 2002) strongly suggested that the TMVB and the adjacent Pacific Ocean constituted a strong dispersal barrier for poeciliids. The present results suggest that this barrier is somewhat “leaky.”

Our analyses of multiple genes from *P.* new species, endemic to the Rio Concepcion, clearly place it within the *Leptorhaphis* complex, possibly as sister to *P. occidentalis s.l.* + *P. lucida* + *P. prolifica*. Only two other freshwater fish genera are native to the Rio Concepcion: *Gila* and *Agosia *(Miller et al., 2005). *Poeciliopsis* new species shares a similar distribution with *Gila ditaenia*, a species endemic to the Rio Concepcion that is highly divergent from other *Gila* spp. examined to date (Schönhuth et al., 2014). The closest sister to *G. ditaenia* has not been identified, but members of the genus inhabit neighboring drainages. This suggests that parts of the Concepcion drainage might have been isolated from other drainages for long periods, enabling the allopatric divergence of *P. *new sp. and *Gila ditaenia* from their respective closest relatives. Because *Gila* species from Mexico are allopatric, Schönhuth et al. (2014) suggest they have likely diverged in allopatry in this arid region. The presence of *P. occidentalis* and the asexual biotype *P. monacha‐occidentalis* in the Concepcion, however, implies relatively recent connections to other drainages (Quattro, Avise, & Vrijenhoek, 1992a). The distribution of *Agosia chrysogaster *in the Concepcion, Sonoyta, and Gila drainages is also consistent with recent connections among these drainages, but genetic characterization of these populations is needed to adequately assess their phylogeographic history. The absence of *P.* new species and *G. ditaenia* from neighboring drainages despite apparently recent drainage connections suggests an ecological constraint prevents their dispersal to, or establishment in, other areas.

### 4.2 Further taxonomic and conservation considerations

*Poeciliopsis balsas* appears to have a disjunct distribution (Figure 2): eastern Balsas, western Balsas (source of our specimen) and two independent smaller rivers (Arteaga and Aguililla) that drain directly to the Pacific. Given their apparent geographic isolation, disjunct populations of *P. balsas* may represent evolutionary independent lineages. Geographic restriction to the eastern Balsas or subdivision between the eastern and other parts of the Balsas drainage has been documented in several freshwater fishes: *Ilyodon* (Beltrán‐López et al., 2017), *Astyanax* (Ornelas‐García et al., 2008), and *Notropis boucardi* (Schönhuth & Doadrio, 2003). Unfortunately, *P. balsas* has been extirpated in at least part of its eastern Balsas range (e.g., Chontalcoatlan‐Amacuzac hydrological system, Morelos and Guerrero), where multiple invasive species have become established, including its congener *P. gracilis *(Mejía‐Mojica, Contreras‐MacBeath, & Ruiz‐Campos, 2015). According to collection records from the Ichthyological collection of the Universidad Michoacana, surveys conducted in 2017–18 indicate that local extirpation occurred in eight of the 10 sites with pre‐2010 historical record (Supporting information Table S4). The two sites where *P. balsas* has been recorded recently are Puente El Marquez, near town of El Chauz, Michoacan (black square in Figure 2) and Ajuchitan del Progreso, Guerrero (white square in Figure 2). Given this, we consider that *P. balsas* must to be listed as an endangered species and that efforts are urgently needed to preserve these populations and to identify additional localities where it may persist.

*Poeciliopsis scarlli* (type locality near Lazaro Cardenas, Michoacan; yellow star in Figure 4) was synonymized with *P. turrubarensis* by Miller et al. (2005). We consider, however, that the degree of genetic divergence observed between specimens we assigned to *P. scarlli* (immediately north and south of the TMVB) and specimens of *P. turrubarensis* (type locality in Costa Rica; orange star in Figure 4) from Central America (uncorrected p distance = 6.45% at the mitochondrial genes; and 0.55% at the nuclear genes) is commensurate with species‐level recognition. The exact distribution limits of the two species and the degree of subdivision within them are yet to be explored with molecular markers. Furthermore, the taxonomic status of Colombian populations with affinity to *P. turrubarensis* (i.e., *Poeciliopsis colombiana* Eigenmann and Henn) has not been adequately assessed (Miller et al., 2005). Phylogeographic breaks (with ~2% divergence at the Cytb gene) are reported within *P. turrubarensis* from different regions of Costa Rica (Larson et al., 2012). One source of taxonomic confusion likely stems from the use by Meyer et al. (1985) of a specimen from San Blas incorrectly assigned to *P. presidionis* for comparison against *P. scarlli* from the type locality and *P. turrubarensis* from Costa Rica. Gonopodial structure of the above specimens was similar, leading Meyer et al. (1985) to propose that *P. presidionis*, *P. scarlli,* and *P. turrubarensis* were part of the “*turrubarensis* complex.” On the basis of a mitochondrial phylogeny, Mateos et al. (2002) moved *P. presidionis* into a separate group containing only *P. presidionis* and its sister taxon *P. turneri*. In addition to our multilocus results indicating that specimens from San Blas are not closely related to *P. presidionis*, Miller et al. (2005) also indicated that populations assigned to *P. presidionis* from south of Rio Acaponeta to San Blas were of “unknown taxonomic status.” Similarly, the estimated degree of maternal provisioning of specimens assigned to *P. presidionis* (i.e., UMMZ 173770 and 173,776) from this region was much lower than those estimated for *P. presidionis* (D.N. Reznick, pers. comm.) in its recognized range (sensu Miller et al., 2005). A diagnostic trait of *P. scarlli *(sensu Meyer et al., 1985) that is only visible in live or fresh specimens; that is, blue rings around the eyes was present in the specimens from San Blas and Cihuatlan (Jalisco) used in our study. Whether this trait is shared with *P. turrubarensis* has not been reported.

The existence of an undescribed sexual *Poeciliopsis* species in the Rio Concepcion, within the range of *P. occidentalis* and the asexual hybrid biotype *P. monacha‐occidentalis*, was suspected since the early 1980 s (R. C. Vrijenhoek, personal communication) and was the subject of two unpublished theses (Sanjur, 1998; Schenk, 1992). The allozyme profile of *P.* new species was interpreted as a mosaic of alleles derived from *P. monacha *and *P. occidentalis* plus uniquely derived alleles. This inference led to the hypothesis that *P.* new sp. originated from the “breakdown” (i.e., return to sexual reproduction) of the sympatric asexual form *P. monacha‐occidentalis *(Schenk, 1992; Vrijenhoek, 1989). Nonetheless, our multilocus results and those of Sanjur (1998; based on Cytb DNA sequences) reveal that the mitochondrial genome of *P.* new sp. is not derived from either *P. monacha* (the maternal progenitor of all asexual forms of *Poeciliopsis*; Mateos & Vrijenhoek, 2002, 2005; Quattro, Avise, & Vrijenhoek, 1991; Quattro et al., 1992a; Quattro, Avise, & Vrijenhoek, 1992b) or *P. occidentalis *(the paternal progenitor of *P. monacha‐occidentalis*), thereby rejecting the “asexual breakdown” hypothesis. More complex hybridization/introgression scenarios for the origin of *P.* new species involving other members of the *Leptorhaphis* complex (clade C) cannot be ruled out with the present data (see green rows in Supporting information Table S3; including concordance factors ranging from 13% to 28%), but neither the nuclear nor the mitochondrial genes examined herein suggest introgression from the *P. monacha* gene pool. It is possible that the allozyme profile of *P.* new sp. is simply the result of shared ancestral polymorphisms and/or convergence (at least based on the crude measure of protein electrophoretic mobility). This system is further complicated by the sympatric occurrence of individuals harboring allozyme profiles consistent with *P. monacha* × *P.* new species hybrids (Schenk, 1992) and mitochondria from *P. monacha* (M. Mateos, personal observation), which may turn out to be one more asexual hybridogenetic form; dependent on fertilization by sperm from *P.* new species rather than *P. occidentalis*. Description of *P.* new sp. is in progress and is a prerequisite for its inclusion in the Mexican list of protected species. Given its restricted distribution to just a few marshy localities in the Rio Concepcion, some of which have been highly modified or destroyed, we consider that this species merits a protection status. Although formal quantification of its population densities awaits, its abundance relative to *P. occidentalis* and the hybrids appears to be very low. Exploration of other potentially suitable habitats for *P.* new sp. may greatly benefit its conservation.

## 5 CONCLUSION

The present multilocus study uncovered the phylogenetic affinities of several members of the genus *Poeciliopsis*. The population of the San Blas area (immediately north of the TMVB) previously assigned to *P. presidionis* but deemed of uncertain taxonomic status by Miller et al. (2005) is closely related to specimens from immediately south of the TMVB assigned to *P. scarlli* by Mateos et al. (2002) (synonymized with *P. turrubarensis* by Miller et al., 2005). This result implies relatively recent exchange across this otherwise apparently strong dispersal barrier. A large sampling gap exits for *P. scarlli*‐*P. turrubarensis* between central Mexico and Costa Rica, and south of Panama, where additional phylogenetic breaks may exist. *Poeciliopsis balsas* represents the only lineage in the “Predominantly Northern” clade that occurs south of the TMVB, but its exact phylogenetic position is unclear. *Poeciliopsis* new species belongs to the *Leptorhaphis *clade and exhibits no evidence of introgression from the *P. monacha* lineage (i.e., the maternal progenitor of all asexual *Poeciliopsis* forms), but genetic exchange with other members of the *Leptorhaphis *clade cannot be ruled out with the present data. Preliminary evidence for one or more reticulation events within the genus (hybrid edges) was detected, but examination of many more markers and multiple individuals per species is needed to better address this and rule out ILS. There is compelling evidence, however, for an admixed genome in *P. retropinna *(subgenus *Aulophallus*)*. *The admixture, sympatry, and high placentation (Reznick et al., 2002) features of the subgenus *Aulophallus* may provide a valuable data point in the quest for understanding the roles of both, maternal provisioning via intergenomic conflict (Crespi & Nosil, 2013; Crespi & Semeniuk, 2004; Furness, Morrison, Orr, Arendt, & Reznick, 2015) and hybridization (reviewed by Schumer, Rosenthal, & Andolfatto, 2018), in promoting speciation. Another species with high placentation whose evolutionary history might have involved reticulation is *P. prolifica* (also a member of the *Leptorhaphis* complex). Clearly the addition of many more loci is necessary to resolve the evolutionary history of this genus and strengthen its value as a system for macro‐evolutionary questions, which should take into account reticulate histories when inferring the mode and tempo of evolution of traits of interest (see Wu, Kostyun, Hahn, & Moyle, 2017). We consider that *P. balsas* and *P.* new species should be protected.

## CONFLICT OF INTEREST

None declared.

## **AUTHOR** **CONTRIBUTIONS**

MM conceived and designed, analyzed, prepared figures and tables, and drafted manuscript. MM ODD AVR contributed materials/data, interpreted results, and reviewed and edited manuscript.

## Data Availability

DNA sequences: sequences generated in this study have been deposited under GenBank Accession Numbers: MH118098‐MH118114 (see Supporting information Table S1). The following files have been deposited in DataDryad https://doi.org/10.5061/dryad.s0b85c2 Dataset** S1.** DNA sequence alignment for the “All genes” dataset. DNA sequence alignment in nexus format and annotated by gene. To generate the “Nuclear only” dataset, the Cytb and ND2 datasets (i.e. “MT only”) were deleted. Taxon labels correspond to “Short Label” column in Table S1. **Dataset S2.** Input file for the *BEAST analyses of the “All genes” dataset in XML format. This is the output generated by the Beauti program. **Dataset S3.** Input file for the *BEAST analyses of the “Nuclear genes” dataset in XML format. This is the output generated by the Beauti program. **Dataset S4.** Input file for the PartitionFinder analyses. **Dataset S5.** Compressed folder including the scripts and intermediate files used to run PhyloNetworks and process its output. **Dataset S6.** KMZ‐formatted file used to generate maps with sampled localities and species distribution polygons. **testAullophalus**. Input and output of R script to run simulation to estimate probability of reticulation within the subgenus *Aullophalus*.
