# Supplementary figures and images for: A multilocus phylogeny of the fish genus Poeciliopsis: Solving taxonomic uncertainties and preliminary evidence of reticulation
Source: Ecol Evol. 2019 Jan 25;9(4):1845–57. doi: 10.1002/ece3.4874 (PMC6392363; doi:10.1002/ece3.4874)

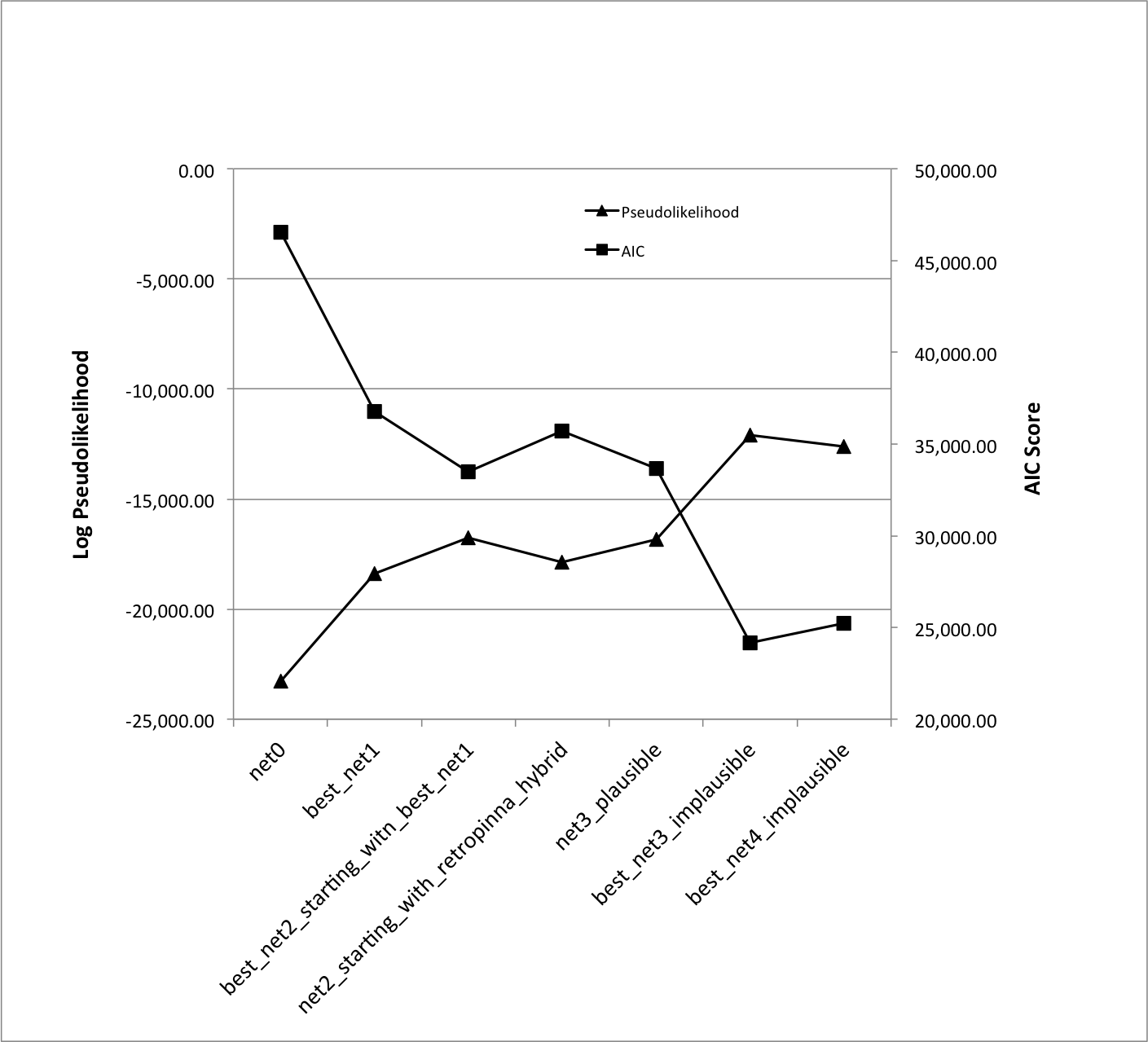

Supplement: Supplementary file 1 [file ECE3-9-1845-s001.png]

# PhyloPlots

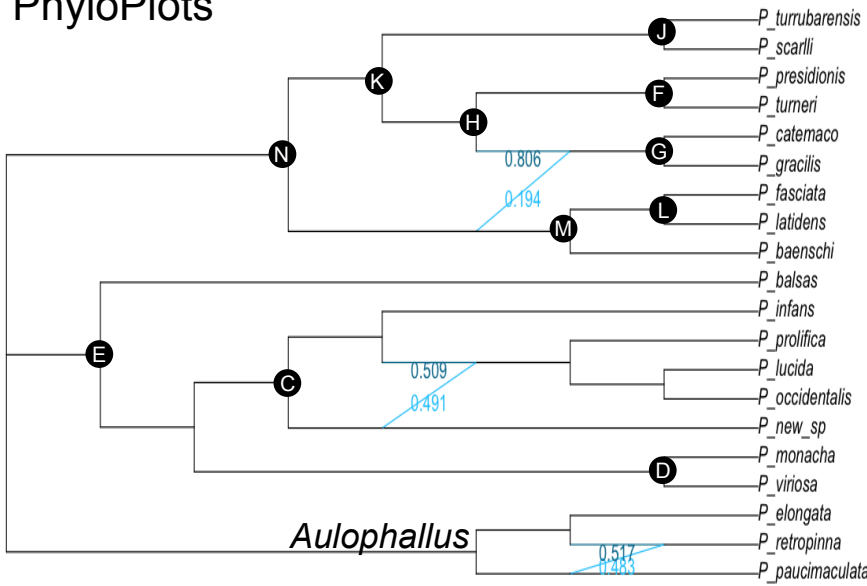

# PhyloPlots

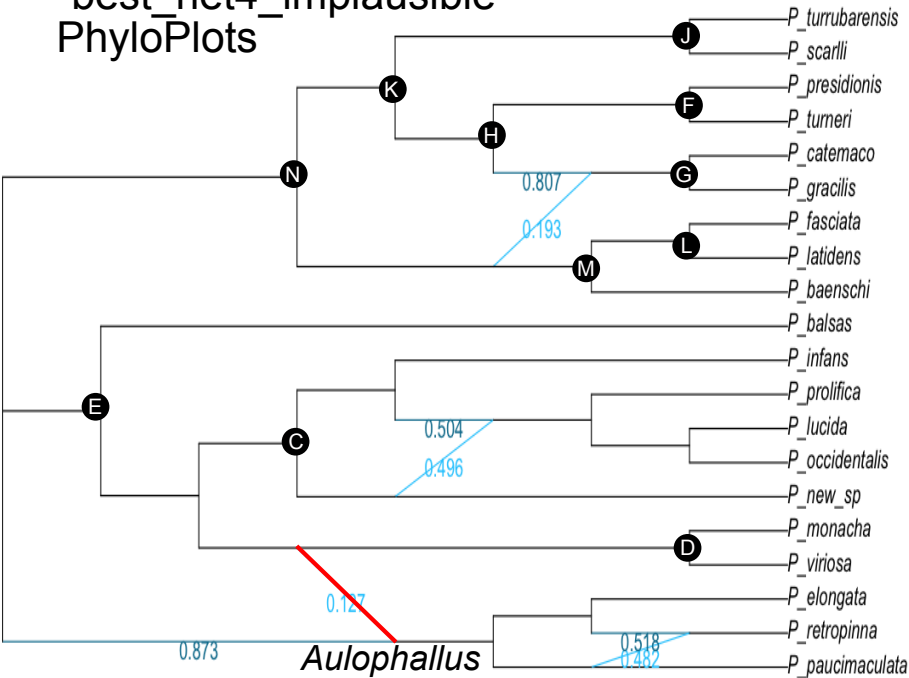

# Dendroscope

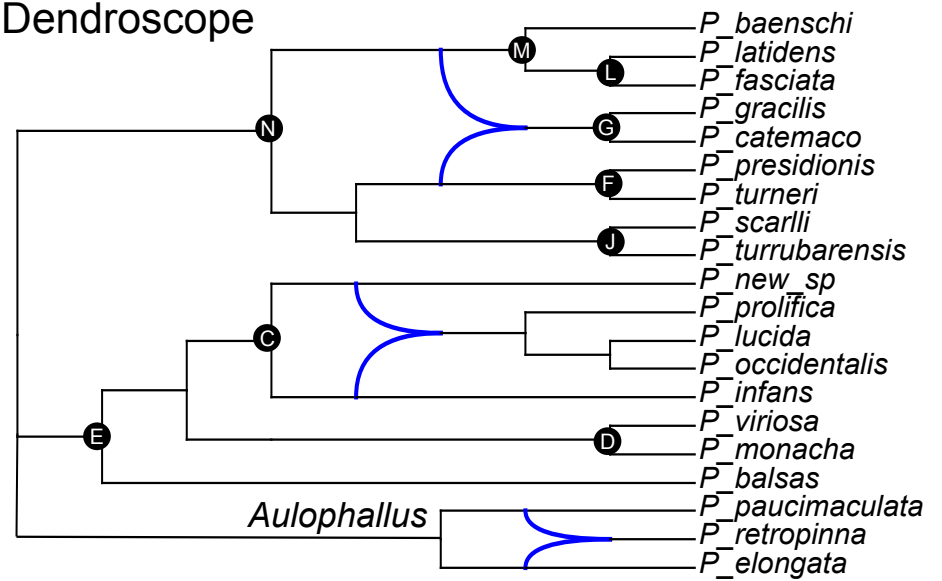

# Dendroscope

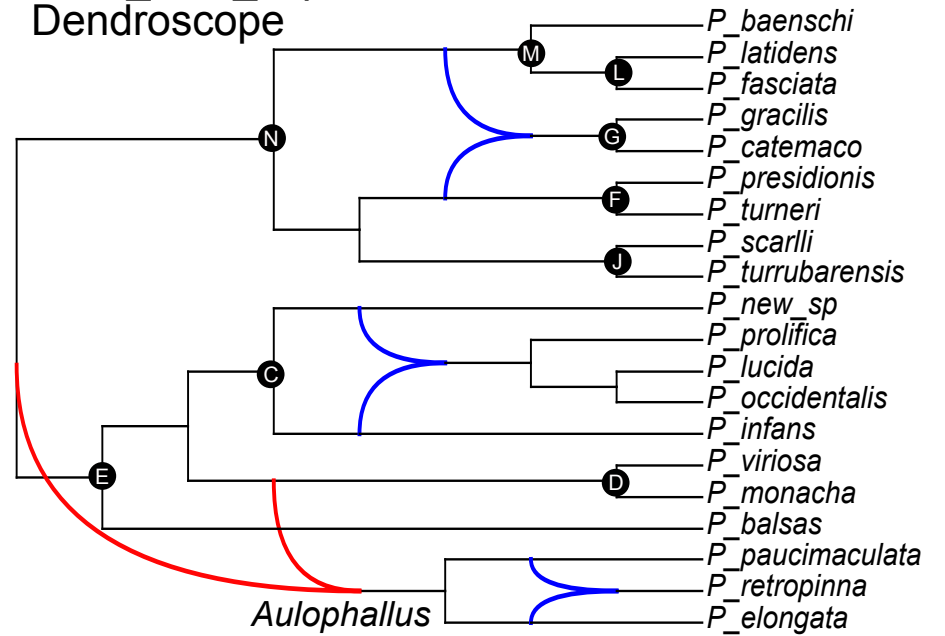

Supplement: Supplementary file 4 [file ECE3-9-1845-s004.pdf]

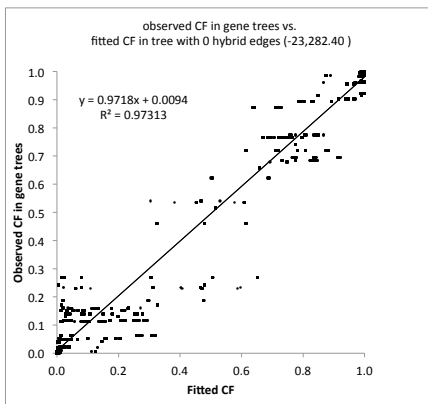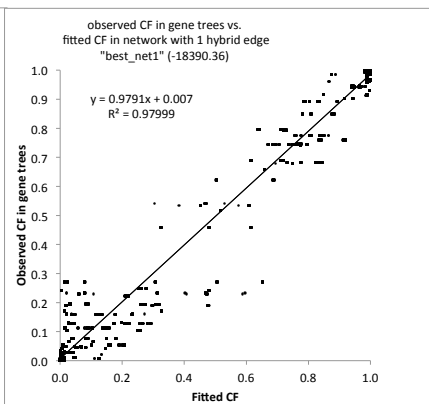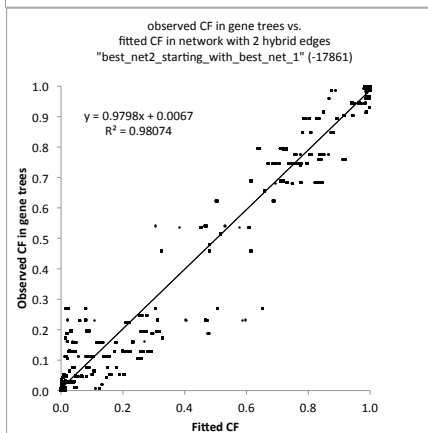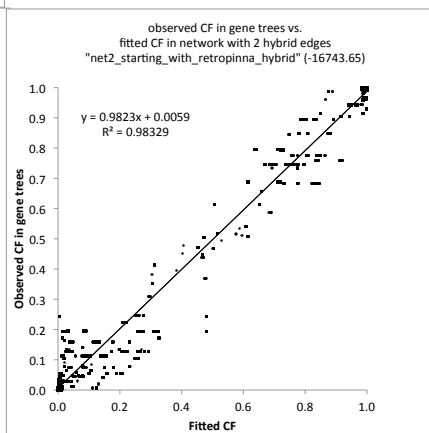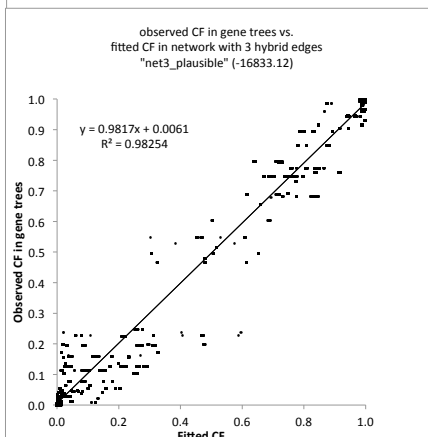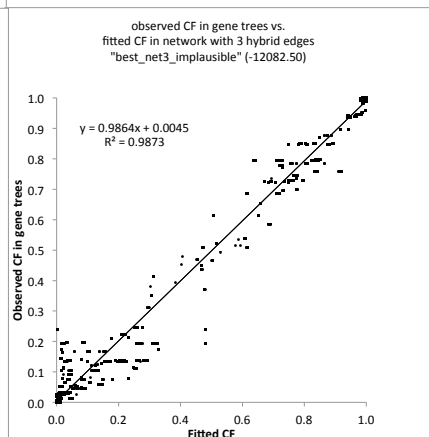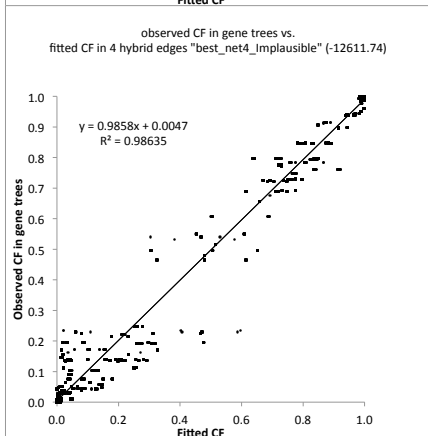

Supplement: Supplementary file 5 [file ECE3-9-1845-s005.pdf]
