# Supplementary material for: A multilocus phylogeny of the fish genus Poeciliopsis: Solving taxonomic uncertainties and preliminary evidence of reticulation
Source: Ecol Evol. 2019 Jan 25;9(4):1845–57. doi: 10.1002/ece3.4874 (PMC6392363; doi:10.1002/ece3.4874)
Supplement: Supplementary file 2 [file ECE3-9-1845-s002.pdf]

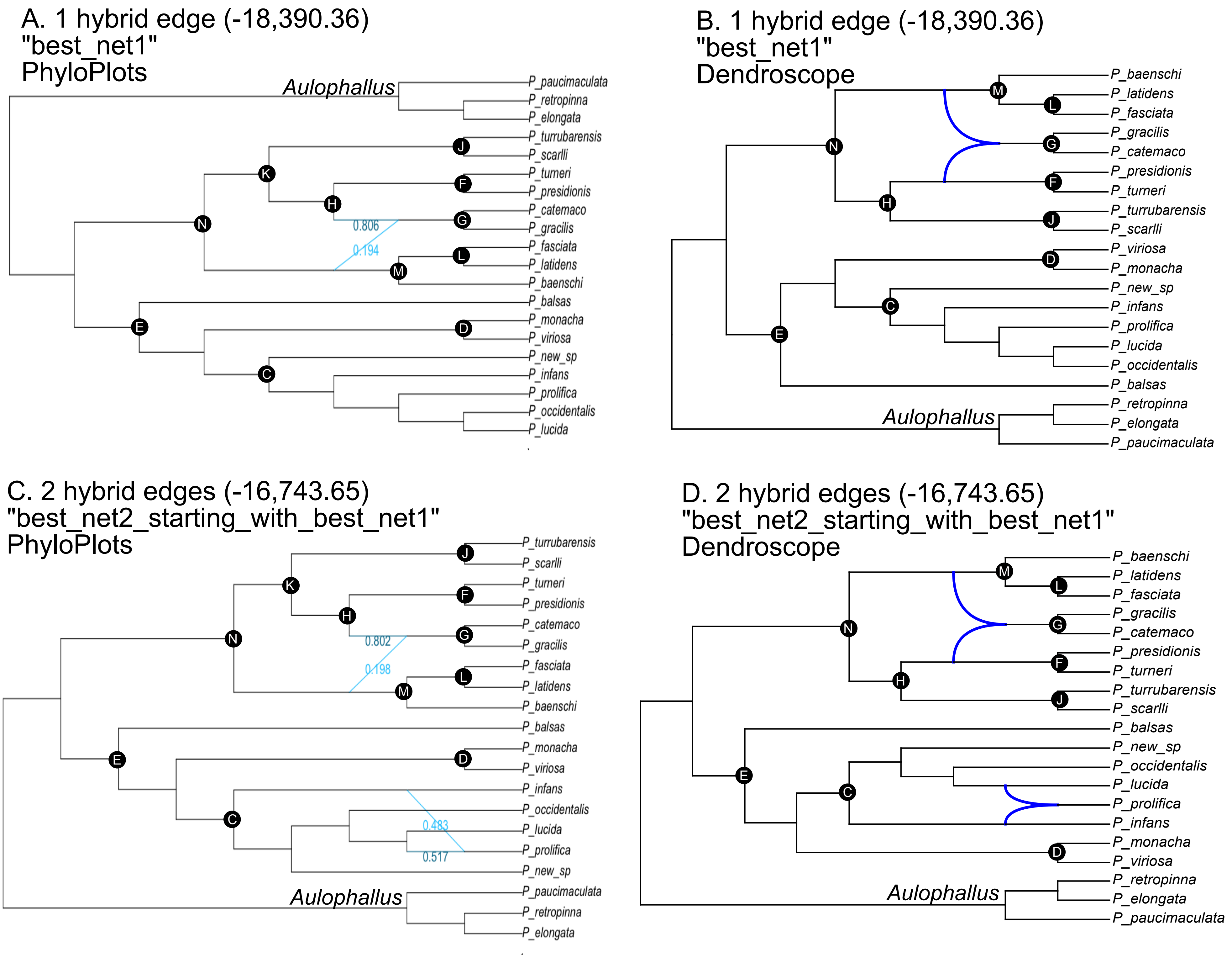
A. 1 hybrid edge (-18,390.36)
"best_net1"
PhyloPlots
Aulophallus
P_paucimaculata
P_retropinna
P_elongata
P_turrubarensis
P_scarlli
P_turneri
P_presidionis
P_catemaco
P_gracilis
P_fasciata
P_latidens
P_baenschi
P_balsas
P_monacha
P_viriosa
P_new_sp
P_infans
P_prolifica
P_occidentalis
P_lucida
J
K
F
H
N
G
L
M
E
D
C
0.806
0.194
B. 1 hybrid edge (-18,390.36)
"best_net1"
Dendroscope
P_baenschi
P_latidens
P_fasciata
P_gracilis
P_catemaco
P_presidionis
P_turneri
P_turrubarensis
P_scarlli
P_viriosa
P_monacha
P_new_sp
P_infans
P_prolifica
P_lucida
P_occidentalis
P_balsas
Aulophallus
P_retropinna
P_elongata
P_paucimaculata
M
L
N
G
F
H
J
D
C
E
C. 2 hybrid edges (-16,743.65)
"best_net2_starting_with_best_net1"
PhyloPlots
P_turrubarensis
P_scarlli
P_turneri
P_presidionis
P_catemaco
P_gracilis
P_fasciata
P_latidens
P_baenschi
P_balsas
P_monacha
P_viriosa
P_infans
P_occidentalis
P_lucida
P_prolifica
P_new_sp
Aulophallus
P_paucimaculata
P_retropinna
P_elongata
J
K
F
H
N
G
L
M
E
D
C
0.802
0.198
0.483
0.517
D. 2 hybrid edges (-16,743.65)
"best_net2_starting_with_best_net1"
Dendroscope
P_baenschi
P_latidens
P_fasciata
P_gracilis
P_catemaco
P_presidionis
P_turneri
P_turrubarensis
P_scarlli
P_balsas
P_new_sp
P_occidentalis
P_lucida
P_prolifica
P_infans
P_monacha
P_viriosa
Aulophallus
P_retropinna
P_elongata
P_paucimaculata
M
L
N
G
F
H
J
E
C
D
