# Supplementary material for: A multilocus phylogeny of the fish genus Poeciliopsis: Solving taxonomic uncertainties and preliminary evidence of reticulation
Source: Ecol Evol. 2019 Jan 25;9(4):1845–57. doi: 10.1002/ece3.4874 (PMC6392363; doi:10.1002/ece3.4874)
Supplement: Supplementary file 3 [file ECE3-9-1845-s003.pdf]

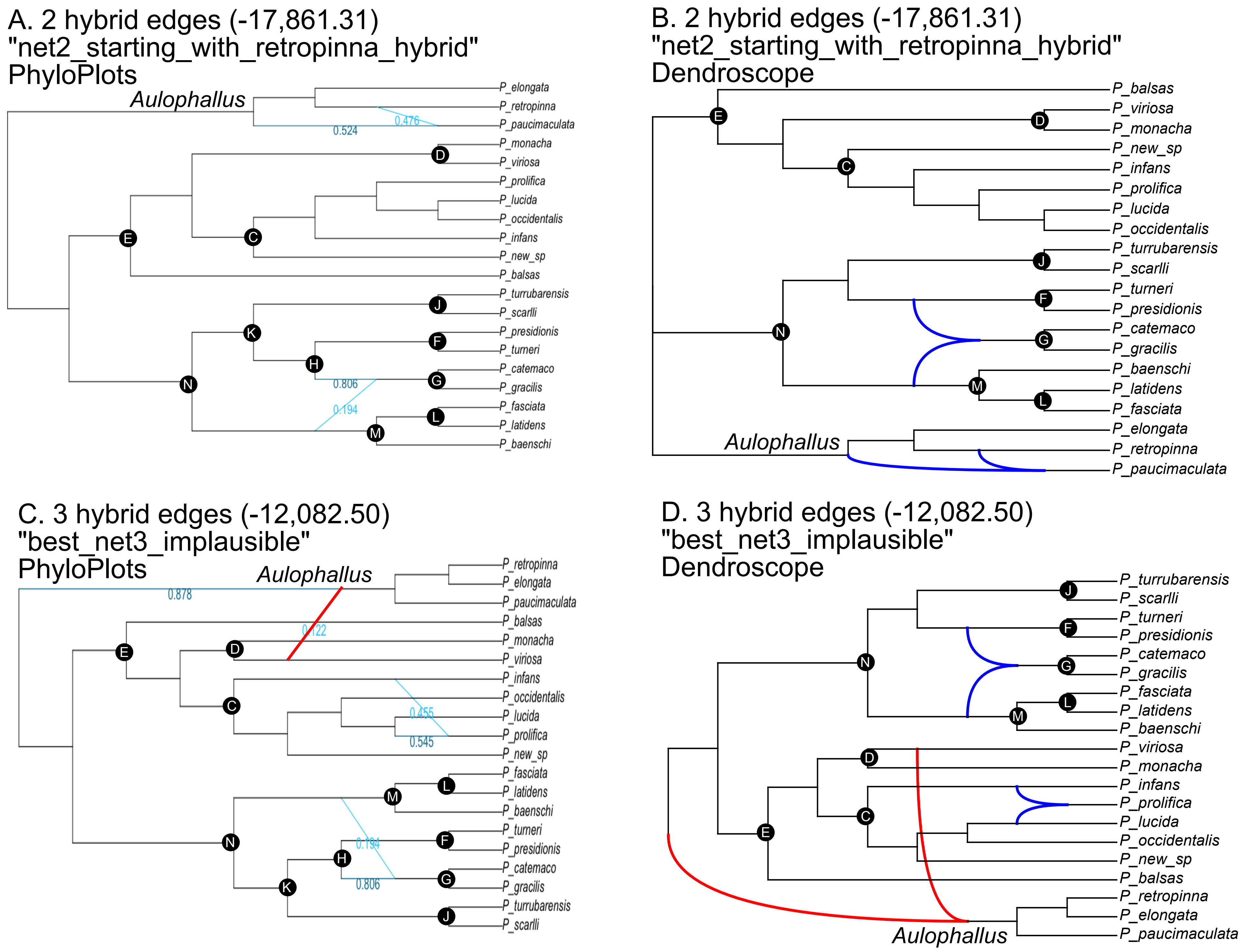
A. 2 hybrid edges (-17,861.31)
"net2_starting_with_retropinna_hybrid"
PhyloPlots
Aulophallus
P_elongata
P_retropinna
P_paucimaculata
0.476
0.524
P_monacha
P_viriosa
P_prolifica
P_lucida
P_occidentalis
P_infans
P_new_sp
P_balsas
P_turrubarensis
P_scarlli
P_presidionis
P_turneri
P_catemaco
P_gracilis
P_fasciata
P_latidens
P_baenschi
0.806
0.194
B. 2 hybrid edges (-17,861.31)
"net2_starting_with_retropinna_hybrid"
Dendroscope
P_balsas
P_viriosa
P_monacha
P_new_sp
P_infans
P_prolifica
P_lucida
P_occidentalis
P_turrubarensis
P_scarlli
P_turneri
P_presidionis
P_catemaco
P_gracilis
P_baenschi
P_latidens
P_fasciata
Aulophallus
P_elongata
P_retropinna
P_paucimaculata
C. 3 hybrid edges (-12,082.50)
"best_net3_implausible"
PhyloPlots
Aulophallus
P_retropinna
P_elongata
P_paucimaculata
0.878
P_balsas
0.122
P_monacha
P_viriosa
P_infans
P_occidentalis
0.455
P_lucida
P_prolifica
0.545
P_new_sp
P_fasciata
P_latidens
P_baenschi
P_turneri
0.194
P_presidionis
P_catemaco
0.806
P_gracilis
P_turrubarensis
P_scarlli
D. 3 hybrid edges (-12,082.50)
"best_net3_implausible"
Dendroscope
P_turrubarensis
P_scarlli
P_turneri
P_presidionis
P_catemaco
P_gracilis
P_fasciata
P_latidens
P_baenschi
P_viriosa
P_monacha
P_infans
P_prolifica
P_lucida
P_occidentalis
P_new_sp
P_balsas
P_retropinna
P_elongata
P_paucimaculata
Aulophallus
