# Supplementary material for: A multilocus phylogeny of the fish genus Poeciliopsis: Solving taxonomic uncertainties and preliminary evidence of reticulation
Source: Ecol Evol. 2019 Jan 25;9(4):1845–57. doi: 10.1002/ece3.4874 (PMC6392363; doi:10.1002/ece3.4874)
Supplement: Supplementary file 6 [file ECE3-9-1845-s006.docx]

Table S1. GenBank accession numbers for sequences used in this study listed by gene.

| **Short Label** | **Genus** | **species** | **voucher** | **Cytb** | **ND2** | **X-src** | **ENC1** | **Glyt** | **Myh6** | **Rag1** | **RH** | **SH3** |
| --- | --- | --- | --- | --- | --- | --- | --- | --- | --- | --- | --- | --- |
| Poecibaen | *Poeciliopsis* | *baenschi* |  | AF412148 | AF412191 | KJ697640 | KJ696941 | KJ697051 | KJ697161 | KJ697324 | KJ697424 | KJ697534 |
| Poecibals | *Poeciliopsis* | *balsas* |  | KJ696842 | KJ697238 | KJ697641 | KJ696942 | KJ697052 | KJ697162 | KJ697325 | KJ697425 | KJ697535 |
| Poecicate | *Poeciliopsis* | *catemaco* |  | AF412161 | AF412201 | KJ697642 | KJ696943 | KJ697053 | KJ697163 | KJ697326 | KJ697426 | KJ697536 |
| Poecielon | *Poeciliopsis* | *elongata* |  | AF412129 | AF412172 | KJ697643 | KJ696944 | KJ697054 | KJ697164 | KJ697327 | KJ697427 | KJ697537 |
| Poecifasc | *Poeciliopsis* | *fasciata* | Coa | AF412149 | AF412193 | KJ697644 | KJ696945 | KJ697055 | KJ697165 | EF017443 | KJ697428 | KJ697538 |
| Poecigrac | *Poeciliopsis* | *gracilis* | TB | AF412155 | AF412195 | KJ697645 | KJ696946 | KJ697056 | KJ697166 | KJ697328 | KJ697429 | KJ697539 |
| Poeciinfa | *Poeciliopsis* | *infans* | Pan | AF412138 | AF412183 | KJ697646 | KJ696947 | KJ697057 | KJ697167 | KJ697329 | KJ697430 | KJ697540 |
| Poecilati | *Poeciliopsis* | *latidens* | Nay | AF412151 | AF412194 | KJ697647 | KJ696948 | KJ697058 | KJ697168 | KJ697330 | KJ697431 | KJ697541 |
| Poeciluci | *Poeciliopsis* | *lucida* |  | AF412139 | AF412184 | KJ697648 | KJ696949 | KJ697059 | KJ697169 | KJ697331 | KJ697432 | KJ697542 |
| Poecimona | *Poeciliopsis* | *monacha* | hap f | AF047346 | AF412173 | KJ697649 | KJ696950 | KJ697060 | KJ697170 | KJ697332 | KJ697433 | KJ697543 |
| Poeciocci | *Poeciliopsis* | *occidentalis* | Alt | AF412141 | AF412185 | KJ697650 | KJ696951 | KJ697061 | KJ697171 | KJ697333 | KJ697434 | KJ697544 |
| Poecipauc | *Poeciliopsis* | *paucimaculata* |  | AF412128 | AF412171 | KJ697651 | KJ696952 | KJ697062 | KJ697172 | KJ697334 | KJ697435 | KJ697545 |
| Poecipres | *Poeciliopsis* | *presidionis* |  | AF412157 | AF412196 | KJ697652 | KJ696953 | KJ697063 | KJ697173 | KJ697335 | KJ697436 | KJ697546 |
| Poeciprol | *Poeciliopsis* | *prolifica* |  | AF412146 | AF412189 | KJ697653 | KJ696954 | KJ697064 | KJ697174 | KJ697336 | KJ697437 | KJ697547 |
| Poeciretr | *Poeciliopsis* | *retropinna* |  | AF412130 | KJ697239 | KJ697654 | KJ696955 | KJ697065 | KJ697175 | KJ697337 | KJ697438 | KJ697548 |
| Poeciscar | *Poeciliopsis* | *scarlli* | South | AF412159 | AF412198 | KJ697655 | KJ696956 | KJ697066 | KJ697176 | KJ697338 | KJ697439 | KJ697549 |
| Poeciturn | *Poeciliopsis* | *turneri* |  | AF412158 | AF412197 | KJ697656 | KJ696957 | KJ697067 | KJ697177 | KJ697339 | KJ697440 | KJ697550 |
| Poeciturr | *Poeciliopsis* | *turrubarensis* | Pan | AF412164 | AF412204 | KJ697657 | KJ696958 | KJ697068 | KJ697178 | KJ697340 | KJ697441 | KJ697551 |
| Poeciviri | *Poeciliopsis* | *viriosa* |  | AF412133 | AF412175 | KJ697658 | KJ696959 | KJ697069 | KJ697179 | KJ697341 | KJ697442 | KJ697552 |
| Pnewspeci | *Poeciliopsis* | *new species* | MVH99-2a#5 | MH118112 | MH118114 | MH118098 | MH118110 | MH118108 | MH118104 | MH118102 | MH118100 | MH118106 |
| PoscarNor | *Poeciliopsis* | *scarlli* | MR04-2#1 (North) | MH118111 | MH118113 | na | MH118109 | MH118107 | MH118103 | MH118101 | MH118099 | MH118105 |
