# Supplementary material for: A multilocus phylogeny of the fish genus Poeciliopsis: Solving taxonomic uncertainties and preliminary evidence of reticulation
Source: Ecol Evol. 2019 Jan 25;9(4):1845–57. doi: 10.1002/ece3.4874 (PMC6392363; doi:10.1002/ece3.4874)
Supplement: Supplementary file 7 [file ECE3-9-1845-s007.docx]

Table S2. Model selection results by Dataset.

| Dataset | Best model according to Jmodeltest (single partition) | Single partition Model Implemented in MrBayes | PartitionFinder Output (greedy BIC) |
| --- | --- | --- | --- |
| Concatenated all genes | AIC GTR+I+G BIC GTR+I+G AICc GTR+I+G | see Table S3 | Subset \| Best Model \| # sites \| subset id \| Partition names  1 \| K80+I+G \| 380 \| 8cb193871c8868696ec7422e24c75538 \| cytb_1  2 \| HKY+I \| 654 \| a0f6a6641ae9e7077832d113b3ee8ac2 \| cytb_2, Rh_1  3 \| TRN+I+G \| 729 \| 53e31c45ed19e9563ed6bc3eb38c2bd3 \| cytb_3, nd2_3  4 \| TRN+I \| 349 \| 4bf8ef9bc1e401559164beffd367f1d5 \| nd2_1  5 \| HKY+G \| 449 \| b6d42515d5f85c05115e3eb167a2246c \| xrsc_intron2, nd2_2  6 \| K80+I \| 1134 \| abf39bd27c06963bc537b9356f6c7c4e \| xsrc10_codon_2, Glyt_1, Rag1_1, ENC_1  7 \| HKY+I \| 1950 \| 6b5aa1e717fb92010862f7ee7cfd782f \| Rh_3, xsrc8_codon_3, xsrc10_codon_3, SH3PX3_2, ENC_2, Myh6_3, Glyt_2, Rag1_2  8 \| HKY+I \| 1702 \| f1938bede056b2f65f59d526be289692 \| xsrc9_codon_1, xsrc10_codon_1, ENC_3, xsrc8_codon_1, Glyt_3, Rag1_3, SH3PX3_3, Myh6_1  9 \| K81UF+G \| 846 \| f73aa63367c7cb8fe3dc3b6e0bc8e7b0 \| Myh6_2, SH3PX3_1, xsrc8_codon_2, Rh_2, xsrc9_codon_3  10 \| JC \| 127 \| 3c4526999bfd38d8c9d0f54ba82dd46a \| xrsc_intron1, xsrc9_codon_2 |
| Only Mt | AIC GTR+I+G BIC TrN+I+G AICc GTR+I+G | see Table S3 | Subset \| Best Model \| # sites \| subset id \| Partition names  1 \| K80+I+G \| 380 \| 8cb193871c8868696ec7422e24c75538 \| cytb_1  2 \| HKY+I \| 380 \| e681de34bfcec5136f8849462851a8b5 \| cytb_2  3 \| TRN+I+G \| 729 \| 53e31c45ed19e9563ed6bc3eb38c2bd3 \| nd2_3, cytb_3  4 \| TRN+I \| 349 \| 4bf8ef9bc1e401559164beffd367f1d5 \| nd2_1  5 \| HKY+G \| 349 \| f5553d729b3ea6516c47714cd76f958a \| nd2_2 |
| Only nuclear | AIC TIM2+I+G BIC TrNef+I+G  AICc TIM2ef+I+G DT TrNef+I+G | see Table S3 | 1 \| K80+G \| 1134 \| b8788155512b31d8a293409c80b62bd2 \| xsrc10_codon_2, Glyt_1, Rag1_1, ENC_1  2 \| HKY+I \| 1676 \| e173b32fff114227d9dbf2d50bef7254 \| SH3PX3_2, xsrc8_codon_3, xsrc10_codon_3, ENC_2, Myh6_3, Glyt_2, Rag1_2  3 \| HKY+I \| 2001 \| cb2d5196edf70f3c2a162c1eeef99fb7 \| xsrc9_codon_1, xsrc10_codon_1, Rh_1, xsrc9_codon_3, Rag1_3, ENC_3, Glyt_3, xsrc8_codon_1, SH3PX3_3, Myh6_1 4 \| K81UF+G \| 821 \| 88e325b02e06c2e28e93cdacdee297d0 \| Rh_2, Myh6_2, SH3PX3_1, xsrc8_codon_2  5 \| TRNEF+I \| 274 \| 4ca449ec5749e9f0f23f942409e63709 \| Rh_3  6 \| HKY \| 227 \| 9fde1acc425316821419c744f45917f6 \| xsrc9_codon_2, xrsc_intron2, xrsc_intron1 |
| ENC | AIC TIM2ef+I BIC TIM2ef+I AICc TIM2ef+I DT TIM2ef+I | lset applyto=(2) nst=6 rates=propinv ;  prset applyto=(2) statefreqpr=fixed(equal); |  |
| Glyt | AIC TIM2+G BIC K80+G AICc TIM2+G DT TPM3+G | lset applyto=(3) nst=2 rates=gamma;  prset applyto=(3) statefreqpr=fixed(equal); |  |
| SH3PX3 | AIC TIM3+I BIC K80+I AICc TIM3+I DT K80+G | lset applyto=(4) nst=2 rates=propinv;  prset applyto=(4) statefreqpr=fixed(equal) |  |
| Myh6 | AIC TrN+I  BIC TrNef+I AICc TrN+I  DT TrNef+G | lset applyto=(5) nst=6 rates=propinv ;  prset applyto=(5) statefreqpr=fixed(equal) |  |
| Rag1 | AIC HKY+I BIC K80+I AICc HKY+I DT K80+I | lset applyto=(6) nst=2 rates=propinv;  prset applyto=(6) statefreqpr=fixed(equal); |  |
| Rh | AIC HKY+I+G BIC HKY+I  AICc HKY+I+G DT HKY+I | lset applyto=(7) nst=2 rates=propinv; |  |
| Xsrc | AIC HKY+G BIC K80  AICc HKY+G DT K80+G | lset applyto=(8) nst=2;  prset applyto=(8) statefreqpr=fixed(equal);prset applyto=(all) ratepr=variable; unlink statefreq=(all) revmat=(all) shape=(all) pinvar=(all) tratio=(all) Topology=(all); |  |
