# Supplementary material for: A multilocus phylogeny of the fish genus Poeciliopsis: Solving taxonomic uncertainties and preliminary evidence of reticulation
Source: Ecol Evol. 2019 Jan 25;9(4):1845–57. doi: 10.1002/ece3.4874 (PMC6392363; doi:10.1002/ece3.4874)
Supplement: Supplementary file 8 [file ECE3-9-1845-s008.docx]

|  | Dataset and Method | | | | | | | | | | | | | | | | | | | | | | | | |
| --- | --- | --- | --- | --- | --- | --- | --- | --- | --- | --- | --- | --- | --- | --- | --- | --- | --- | --- | --- | --- | --- | --- | --- | --- | --- |
|  | MrBayes Individual nuclear genes | | | | | | | Bayesian Concordance Analyses | All genes (mitochondrial and nuclear) | | | | | | | | | | | | | | | | |
| Clade | MrBayes ENC | MrBayes Glyt | MrBayes SH3PX3 | MrBayes Myh6 | MrBayes Rag1 | MrBayes Rh | MrBayes Xsrc | Bucky Concordance Factor % (apha = 1) | RaxML Single Partition | RaxML best scheme partition | IQTree Single Partition (GTR+G+I) SH-aLRT | IQTree Single Partition (GTR+G+I) aBayes | IQTree Single Partition (GTR+G+I) Ultrafast Bootstrap | IQTree Single Partition (model selection) SH-aLRT | IQTree Single Partition (model selectionI) aBayes | IQTree Single Partition (model selection) Standard Bootstrap | IQTree best scheme partition SH-aLRT | IQTree best scheme partition aBayes | IQTree best scheme partition Ultrafast Bootstrap | PhyML (GTR+G+I) Bootstrap | PhyML (GTR+G+I) SH-aLRT | PhyML (GTR+G+I) aBayes | MrBayes GTR+G+I (closest to TIM2+G+I) | MrBayes best scheme partition | **Species Tree (*BEAST)** |
| Subgenus *Poeciliopsis* vs. Subgenus *Aulophallus* | 100 | 100 | 100 | 100 | 100 | 100 | 100 | 100 | 100 | 100 | 100 | 100 | 100 | 100 | 100 | 100 | 100 | 100 | 100 | 100 | 100 | 100 | 100 | 100 | 100 |
| P. retropinna + P. paucimaculata | 92 |  |  | 72 |  | 100 | 78 | 46.8 | 94 | 94 | 72 | 100 | 74 | 69 | 100 | 75 | 80 | 100 | 77 | 80 | 76 | 100 | 100 | 95 |  |
| P. retropinna + P. elongata |  | 80 | 88 |  | 100 |  |  | 49.4 |  |  |  |  |  |  |  |  |  |  |  |  |  |  |  |  | 62 |
| E = "Predominantly Northern" |  |  | 100 | 100 | 100 |  | 94 | 60.6 | 100 | 100 | 100 | 100 | 99 | 100 | 100 | 99 | 100 | 100 | 100 | 100 | 100 | 100 | 100 | 100 | 100 |
| D = P. viriosa + P. monacha |  | 85 |  | 100 | 100 |  | 100 | 76.8 | 100 | 100 | 100 | 100 | 100 | 100 | 100 | 100 | 100 | 100 | 100 | 100 | 100 | 100 | 100 | 100 | 100 |
| C = Leptorhaphis | 86 |  | 100 | 96 | 100 | 100 | 100 | 86.8 | 100 | 100 | 100 | 100 | 100 | 100 | 100 | 100 | 100 | 100 | 100 | 100 | 100 | 100 | 100 | 100 | 100 |
| C + D |  |  | 68 |  |  |  | 96 | 40.6 | 89 | 91 | 88 | 95 | 83 | 87 | 94 | 85 | 90 | 100 | 85 | 79 | 87 | 96 | 99 | 99 |  |
| D + P. balsas |  |  |  |  |  |  |  | 13.2 |  |  |  |  |  |  |  |  |  |  |  |  |  |  |  |  | 80 |
| N + D + P. balsas |  |  |  |  |  |  |  |  |  |  |  |  |  |  |  |  |  |  |  |  |  |  |  |  |  |
| P. prolifica + P. occidentalis |  |  |  |  |  |  |  | 13.5 |  |  |  |  |  |  |  |  |  |  |  |  |  |  |  |  |  |
| P. prolifica + P. lucida |  |  | 100 |  |  |  |  | 27.5 | 98 | 93 | 94 | 100 | 95 | 95 | 100 | 91 | 92 | 100 | 89 | 90 | 93 | 100 | 100 | 100 | 53 |
| A = P. prolifica + P. lucida + P. occidentalis |  |  |  |  |  |  |  | 27.8 | 100 | 97 | 99 | 100 | 100 | 99 | 100 | 95 | 99 | 100 | 92 | 98 | 100 | 100 | 100 | 100 | 98 |
| B = A + P. new sp. | 99 |  |  |  |  |  |  | 26.8 | 89 | 70 | 88 | 100 | 87 | 90 | 100 | 88 | 52 | 96 | 63 | 86 | 87 | 100 | 100 | 100 | 83 |
| P. prolifica + P. infans |  | 100 |  |  | 100 |  |  | 27.2 |  |  |  |  |  |  |  |  |  |  |  |  |  |  |  |  |  |
| P. prolifica + P. infans + P. lucida |  |  | 100 |  |  |  | 93 | 18.3 |  |  |  |  |  |  |  |  |  |  |  |  |  |  |  |  |  |
| P. prolifica + P. infans + P. lucida + P. occidentalis |  |  |  |  | 77 |  | 100 | 24.3 |  |  |  |  |  |  |  |  |  |  |  |  |  |  |  |  |  |
| P. lucida + P. occidentalis |  | 93 |  |  |  |  |  | 26.2 |  |  |  |  |  |  |  |  |  |  |  |  |  |  |  |  |  |
| P. lucida + P. occidentalis + P. new sp. |  | 91 |  |  |  |  |  | 17.3 |  |  |  |  |  |  |  |  |  |  |  |  |  |  |  |  |  |
| P. occidentalis + P. new sp. |  |  | 99 |  |  |  |  | 19.3 |  |  |  |  |  |  |  |  |  |  |  |  |  |  |  |  |  |
| P. infans + P. lucida + P. occidentalis + P. new sp. |  |  |  |  |  | 89 |  | 13.1 |  |  |  |  |  |  |  |  |  |  |  |  |  |  |  |  |  |
| L = P. fasciata + P. latidens |  | 100 | 87 |  |  | 98 |  | 56.8 | 100 | 100 | 100 | 100 | 100 | 100 | 100 | 100 | 100 | 100 | 100 | 100 | 99 | 100 | 100 | 100 | 97 |
| M = L + P. baenschi | 100 | 100 | 100 | 91 | 100 | 99 |  | 98.7 | 100 | 100 | 100 | 100 | 100 | 100 | 100 | 100 | 100 | 100 | 100 | 100 | 100 | 100 | 100 | 100 | 100 |
| P. baenschi + P. fasciata |  |  |  | 100 |  |  |  | 33.1 |  |  |  |  |  |  |  |  |  |  |  |  |  |  |  |  |  |
| I = P. scarlli North + P. scarlli South | n/a | n/a | n/a | n/a | n/a | n/a | n/a | n/a | 100 | 100 | 100 | 100 | 100 | 100 | 100 | 100 | 100 | 100 | 100 | 100 | 100 | 100 | 100 | 100 | n/a |
| J = I + P. turrubarensis | 94 | 100 | 99 | 100 | 100 | 80 | 100 | 96.6 | 100 | 100 | 100 | 100 | 100 | 100 | 100 | 100 | 100 | 100 | 100 | 100 | 100 | 100 | 100 | 100 | 100 |
| N = "Predominantly Southern" = K + M |  | 79 |  |  |  | 84 | 100 | 43.7 | 59 | 59 | 61 | 98 | 68 | 57 | 98 | 57 | 58 | 98 | 59 | 65 | 47 | 90 | 99 | 96 | 100 |
| F = P. presidionis + P. turneri |  |  |  | 89 | 100 | 100 | 96 | 77.3 | 100 | 100 | 100 | 100 | 100 | 100 | 100 | 100 | 100 | 100 | 100 | 100 | 100 | 100 | 100 | 100 | 100 |
| G = P. gracilis + P. catemaco | 97 |  | 89 | 100 | 100 | 90 | 100 | 97 | 100 | 100 | 100 | 100 | 100 | 100 | 100 | 100 | 100 | 100 | 100 | 100 | 100 | 100 | 100 | 100 | 100 |
| H = F + G |  |  |  |  | 100 | 99 |  | 46.4 | 100 | 100 | 100 | 100 | 100 | 100 | 100 | 100 | 100 | 100 | 100 | 100 | 100 | 100 | 100 | 100 | 100 |
| K = H + J |  |  |  |  | 99 | 84 |  | 40.3 | 100 | 100 | 100 | 100 | 100 | 100 | 100 | 100 | 100 | 100 | 100 | 100 | 100 | 100 | 100 | 100 | 98 |
| K + E |  |  |  |  |  |  |  | 7.0 |  |  |  |  |  |  |  |  |  |  |  |  |  |  |  |  |  |
| M + G | 86 |  |  |  |  |  | 91 | 23.2 |  |  |  |  |  |  |  |  |  |  |  |  |  |  |  |  |  |
| M + J |  | 54 |  |  |  |  |  | 10.7 |  |  |  |  |  |  |  |  |  |  |  |  |  |  |  |  |  |
| M + E |  |  |  |  | 89 |  |  | 20.2 |  |  |  |  |  |  |  |  |  |  |  |  |  |  |  |  |  |
| C + N |  |  |  |  |  | 75 |  | <5.0 |  |  |  |  |  |  |  |  |  |  |  |  |  |  |  |  |  |
| C + D + N (=subgenus Poeciliopsis excluding P. balsas) |  |  |  |  |  | 95 |  | 13.6 |  |  |  |  |  |  |  |  |  |  |  |  |  |  |  |  |  |
| subgenus Poeciliopsis excluding P. viriosa | 99 |  |  |  |  |  |  | 12.6 |  |  |  |  |  |  |  |  |  |  |  |  |  |  |  |  |  |
| subgenus Poeciliopsis excluding P. viriosa and P. monacha | 91 |  |  |  |  |  |  | 12.7 |  |  |  |  |  |  |  |  |  |  |  |  |  |  |  |  |  |
