# Supplementary material for: A multilocus phylogeny of the fish genus Poeciliopsis: Solving taxonomic uncertainties and preliminary evidence of reticulation
Source: Ecol Evol. 2019 Jan 25;9(4):1845–57. doi: 10.1002/ece3.4874 (PMC6392363; doi:10.1002/ece3.4874)
Supplement: Supplementary file 9 [file ECE3-9-1845-s009.docx]

|  | Dataset and Method | | | | | | | | | | | | | | | | | | | | | | | | | | | | | | | |
| --- | --- | --- | --- | --- | --- | --- | --- | --- | --- | --- | --- | --- | --- | --- | --- | --- | --- | --- | --- | --- | --- | --- | --- | --- | --- | --- | --- | --- | --- | --- | --- | --- |
|  | Mitochondrial genes only (MT only) | | | | | | | | | | | | | | Nuclear genes only (Nuclear only) | | | | | | | | | | | | | | | | | |
| Clade | RaxML Single Partition | RaxML best scheme partition | IQTree Single Partition (GTR+G+I) SH-aLRT | IQTree Single Partition (GTR+G+I) aBayes | IQTree Single Partition (GTR+G+I) Ultrafast Bootstrap | IQTree best scheme partition SH-aLRT | IQTree best scheme partition aBayes | IQTree best scheme partition Ultrafast Bootstrap | PhyML (GTR+G+I) Bootstrap | PhyML (GTR+G+I) SH-aLRT | PhyML (GTR+G+I) aBayes | MrBayes GTR+G+I | MrBayes best scheme partition (RUN 2) | MrBayes best scheme partition | RaxML Single Partition | RaxML best scheme partition | IQTree Single Partition (SYM+G+I) SH-aLRT | IQTree Single Partition (SYM+G+I) aBayes | IQTree Single Partition (SYM+G+I) Ultrafast Bootstrap | IQTree best scheme partition SH-aLRT | IQTree best scheme partition aBayes | IQTree best scheme partition Ultrafast Bootstrap | PhyML (GTR+G+I) Bootstrap | PhyML (GTRR+G+I) SH-aLRT | PhyML (GTR+G+I) aBayes | MrBayes SYM+I+G | MrBayes SYM+I+G (no Beagle) | MrBayes GTR+G+I run 2 | MrBayes GTR+G+I | MrBayes best scheme partition | MrBayes best scheme partition (no Beagle) | **Species Tree (*BEAST)** |
| Subgenus *Poeciliopsis* vs. Subgenus *Aulophallus* | 100 | 100 | 100 | 100 | 100 | 100 | 100 | 100 | 100 | 100 | 100 | 100 | 100 | 100 | 100 | 100 | 100 | 100 | 100 | 100 | 100 | 100 | 100 | 100 | 100 | 100 | 100 | 100 | 100 | 100 | 100 | 100 |
| P. retropinna + P. paucimaculata | 97 | 98 | 94 | 100 | 93 | 98 | 100 | 97 | 90 | 95 | 100 | 100 | 100 | 100 |  |  | 60 | 98 | 77 |  |  |  |  |  |  | 91 |  | 86 | 86 |  |  |  |
| P. retropinna + P. elongata |  |  |  |  |  |  |  |  |  |  |  |  |  |  | 78 | 98 |  |  |  | 93 | 100 | 91 | 65 | 59 | 94 |  | 98 |  |  | 100 | 100 | 67 |
| E = "Predominantly Northern" | 93 | 98 | 93 | 100 | 71 | 99 | 100 | 71 | 79 | 93 | 100 | 82 |  |  | 99 | 99 | 99 | 100 | 98 | 99 | 100 | 98 | 100 | 99 | 100 | 100 | 100 | 100 | 100 | 100 | 100 | 100 |
| D = P. viriosa + P. monacha | 100 | 100 | 100 | 100 | 100 | 100 | 100 | 100 | 100 | 100 | 100 | 100 | 100 | 100 | 100 | 100 | 100 | 100 | 100 | 100 | 100 | 100 | 100 | 100 | 100 | 100 | 100 | 100 | 100 | 100 | 100 | 100 |
| C = Leptorhaphis | 100 | 100 | 100 | 100 | 100 | 100 | 100 | 100 | 100 | 100 | 100 | 100 | 100 | 100 | 100 | 100 | 100 | 100 | 100 | 100 | 100 | 100 | 100 | 100 | 100 | 100 | 100 | 100 | 100 | 100 | 100 | 100 |
| C + D | 82 | 85 | 84 | 87 | 62 | 81 | 90 | 63 | 68 | 85 | 87 | 74 |  |  | 55 | 65 | 31 | 63 | 64 | 74 | 78 | 77 | 64 | 28 | 63 | 68 | 67 |  |  | 87 | 87 | 55 |
| D + P. balsas |  |  |  |  |  |  |  |  |  |  |  |  |  |  |  |  |  |  |  |  |  |  |  |  |  |  |  | 76 | 75 |  |  |  |
| N + D + P. balsas |  |  |  |  |  |  |  |  |  |  |  |  | 56 | 56 |  |  |  |  |  |  |  |  |  |  |  |  |  |  |  |  |  |  |
| P. prolifica + P. occidentalis |  |  |  |  |  |  |  |  |  |  |  |  | 64 | 65 |  |  |  |  |  |  |  |  |  |  |  |  |  |  |  |  |  |  |
| P. prolifica + P. lucida | 52 | 51 | 48 | 55 | 77 | 0 | 38 | 55 | 55 | 61 | 55 | 60 |  |  |  |  |  |  |  |  |  |  | 59 |  | 100 |  |  |  |  |  |  | 57 |
| A = P. prolifica + P. lucida + P. occidentalis | 100 | 100 | 99 | 100 | 100 | 100 | 100 | 100 | 96 | 100 | 100 | 100 | 100 | 100 |  |  |  |  |  |  |  |  | 47 |  | 61 |  |  |  |  |  |  | 75 |
| B = A + P. new sp. | 91 | 77 | 90 | 99 | 90 | 62 | 86 | 75 | 86 | 86 | 99 | 99 | 96 | 96 |  |  |  |  |  |  |  |  |  |  |  |  |  |  |  |  |  |  |
| P. prolifica + P. infans |  |  |  |  |  |  |  |  |  |  |  |  |  |  | 67 | 76 | 70 | 100 | 62 | 68 | 100 | 59 |  | 72 |  | 100 | 100 | 100 | 100 | 100 | 100 |  |
| P. prolifica + P. infans + P. lucida |  |  |  |  |  |  |  |  |  |  |  |  |  |  | 79 | 77 | 95 | 99 | 63 | 83 | 99 | 57 |  | 82 |  | 100 | 100 | 100 | 100 | 100 | 100 |  |
| P. prolifica + P. infans + P. lucida + P. occidentalis |  |  |  |  |  |  |  |  |  |  |  |  |  |  | 89 | 83 | 92 | 100 | 77 | 94 | 100 | 77 | 54 | 90 | 91 | 100 | 100 | 100 | 100 | 100 | 100 | 51 |
| P. lucida + P. occidentalis |  |  |  |  |  |  |  |  |  |  |  |  |  |  |  |  |  |  |  |  |  |  |  |  |  |  |  |  |  |  |  |  |
| P. lucida + P. occidentalis + P. new sp. |  |  |  |  |  |  |  |  |  |  |  |  |  |  |  |  |  |  |  |  |  |  |  |  |  |  |  |  |  |  |  |  |
| P. occidentalis + P. new sp. |  |  |  |  |  |  |  |  |  |  |  |  |  |  |  |  |  |  |  |  |  |  |  |  |  |  |  |  |  |  |  |  |
| P. infans + P. lucida + P. occidentalis + P. new sp. |  |  |  |  |  |  |  |  |  |  |  |  |  |  |  |  |  |  |  |  |  |  |  |  |  |  |  |  |  |  |  |  |
| L = P. fasciata + P. latidens | 99 | 99 | 98 | 100 | 98 | 98 | 100 | 98 | 98 | 98 | 100 | 100 | 100 | 100 | 95 | 94 | 93 | 100 | 96 | 92 | 100 | 96 | 90 | 92 | 100 | 100 | 100 | 100 | 100 | 100 | 100 | 86 |
| M = L + P. baenschi | 100 | 100 | 100 | 100 | 100 | 100 | 100 | 100 | 100 | 100 | 100 | 100 | 100 | 100 | 100 | 100 | 100 | 100 | 100 | 100 | 100 | 100 | 100 | 100 | 100 | 100 | 100 | 100 | 100 | 100 | 100 | 100 |
| P. baenschi + P. fasciata |  |  |  |  |  |  |  |  |  |  |  |  |  |  |  |  |  |  |  |  |  |  |  |  |  |  |  |  |  |  |  |  |
| I = P. scarlli North + P. scarlli South | 100 | 100 | 100 | 100 | 100 | 100 | 100 | 100 | 100 | 100 | 100 | 100 | 100 | 100 | 100 | 100 | 99 | 100 | 100 | 100 | 100 | 100 | 100 | 100 | 100 | 100 | 100 | 100 | 100 | 100 |  | n/a |
| J = I + P. turrubarensis | 100 | 100 | 100 | 100 | 100 | 100 | 100 | 100 | 100 | 100 | 100 | 100 | 100 | 100 | 100 | 100 | 100 | 100 | 100 | 100 | 100 | 100 | 100 | 100 | 100 | 100 | 100 | 100 | 100 | 100 | 100 | 100 |
| N = "Predominantly Southern" = K + M |  |  | 22 | 65 | 54 |  |  |  | 51 | 18 | 62 | 62 | 75 | 75 | 73 | 68 | 81 | 99 | 85 | 89 | 100 | 87 | 64 | 81 | 98 | 99 | 98 | 84 | 84 | 100 | 100 | 100 |
| F = P. presidionis + P. turneri | 100 | 100 | 100 | 100 | 100 | 100 | 100 | 100 | 100 | 100 | 100 | 100 | 100 | 100 | 100 | 100 | 100 | 100 | 100 | 100 | 100 | 100 | 100 | 100 | 100 | 100 | 100 | 100 | 100 | 100 | 100 | 100 |
| G = P. gracilis + P. catemaco | 100 | 100 | 100 | 100 | 100 | 100 | 100 | 100 | 100 | 100 | 100 | 100 | 100 | 100 | 100 | 100 | 100 | 100 | 100 | 100 | 100 | 100 | 100 | 100 | 100 | 100 | 100 | 100 | 100 | 100 | 100 | 100 |
| H = F + G | 100 | 100 | 99 | 100 | 100 | 99 | 100 | 100 | 100 | 100 | 100 | 100 | 100 | 100 | 95 | 97 | 93 | 100 | 96 | 92 | 100 | 93 | 93 | 93 | 100 | 100 | 100 | 100 | 100 | 100 | 100 | 92 |
| K = H + J | 100 | 100 | 100 | 100 | 100 | 100 | 100 | 100 | 100 | 100 | 100 | 100 | 100 | 100 | 84 | 83 | 83 | 99 | 81 | 81 | 99 | 80 | 72 | 83 | 99 | 99 | 99 | 99 | 99 | 99 | 99 | 52 |
| K + E | 61 |  |  |  |  | 21 | 67 | 41 |  |  |  |  |  |  |  |  |  |  |  |  |  |  |  |  |  |  |  |  |  |  |  |  |
| M + G |  |  |  |  |  |  |  |  |  |  |  |  |  |  |  |  |  |  |  |  |  |  |  |  |  |  |  |  |  |  |  |  |
| M + J |  |  |  |  |  |  |  |  |  |  |  |  |  |  |  |  |  |  |  |  |  |  |  |  |  |  |  |  |  |  |  |  |
| M + E |  |  |  |  |  |  |  |  |  |  |  |  |  |  |  |  |  |  |  |  |  |  |  |  |  |  |  |  |  |  |  |  |
| C + N |  |  |  |  |  |  |  |  |  |  |  |  |  |  |  |  |  |  |  |  |  |  |  |  |  |  |  |  |  |  |  |  |
| C + D + N (=subgenus Poeciliopsis excluding P. balsas) |  |  |  |  |  |  |  |  |  |  |  |  |  |  |  |  |  |  |  |  |  |  |  |  |  |  |  |  |  |  |  |  |
| subgenus Poeciliopsis excluding P. viriosa |  |  |  |  |  |  |  |  |  |  |  |  |  |  |  |  |  |  |  |  |  |  |  |  |  |  |  |  |  |  |  |  |
| subgenus Poeciliopsis excluding P. viriosa and P. monacha |  |  |  |  |  |  |  |  |  |  |  |  |  |  |  |  |  |  |  |  |  |  |  |  |  |  |  |  |  |  |  |  |
