# Supplementary material for: A multilocus phylogeny of the fish genus Poeciliopsis: Solving taxonomic uncertainties and preliminary evidence of reticulation
Source: Ecol Evol. 2019 Jan 25;9(4):1845–57. doi: 10.1002/ece3.4874 (PMC6392363; doi:10.1002/ece3.4874)
Supplement: Supplementary file 10 [file ECE3-9-1845-s010.docx]

| **Catalogue number** | **Collection Date (“historical”)** | **Locality** | **Coordinates** | **Last visit** | **Status during last visit** |
| --- | --- | --- | --- | --- | --- |
| 517 | 1989 | Presa El Pejo, Michoacán | 18°41’38.22”N, 100°56’52.53”W | 2016 | Extirpated |
| 934 | 1986 | River at La Bejuquera, Michoacán | 18°45’53.4”N, 101°16’23.41”W | 2016 | Extirpated |
| 1714 | 2004 | Stream outside Parácuaro, Michoacán | 19°08’15.73”N, 102°04’48.82”W | 2016 | Extirpated |
| 7746 | 2009 | El Manguito, 2.6 km SW of Presa Cupatitzio’s dam wall | 19°19’4.78”N, 102°04’48.82”W | 2017 | Extirpated |
| 7839 | 2009 | Bridge La Pastoria, 5.7 km NW of town of Nueva Italia, Michoacán | 19°05’31.99”N, 102°04’9.53”W | 2017 | Extirpated |
| T42884 | 2010 | Irrigation channel at Valle de Cajones, Nuevo Urecho, Michoacán | 19°10’26.90”N 101°53’41.63”W | 2018 | Extirpated |
| 14223 | 2010 | Stream near Andrés Figueroa dam, Ajuchitlán del Progreso, Guerrero | 18° 4’55.24”N, 100°30’52.06”W | 2017 | Present |
| T4257 | 2004 | Río El Marquez, near town of El Chauz, Michoacán | 18°51'38.01”N, 102° 3'40.62”W | 2017 | Present |
| T3327 | 2004 | Río El Marquez, near town of Nueva Italia | 19° 0'25.24”N, 102° 3'17.23”W | 2018 | Extirpated |
| Not catalogued | 2002 | Stream under Antúnez to Apatzingán highway | 19° 0'59.92”N, 102°15'11.78”W | 2018 | Extirpated |

**Table S4.** Records of extirpation and persistence of *Poeciliopsis balsas* at ten localities in the states Michoacán and Guerrero. Source: Colección de Peces de la Universidad Michoacana de San Nicolás de Hidalgo (CPUM). Note: these localities do not include the records of extirpation in the Chontalcoatlan-Amacuzac (Morelos and Guerrero) portion of the Rio Balsas (Mejía-Mojica et al., 2015).
